# Real-time estimation and biofeedback of single-neuron firing rates using local field potentials

**DOI:** 10.1038/ncomms6462

**Published:** 2014-11-14

**Authors:** Thomas M. Hall, Kianoush Nazarpour, Andrew Jackson

**Affiliations:** 1 Institute of Neuroscience, Medical School, Newcastle University, Newcastle-upon-Tyne NE2 4HH, UK; 2 School of Electrical and Electronic Engineering, Newcastle University, Newcastle-upon-Tyne NE1 7RU, UK

## Abstract

The long-term stability and low-frequency composition of local field potentials (LFPs) offer important advantages for robust and efficient neuroprostheses. However, cortical LFPs recorded by multi-electrode arrays are often assumed to contain only redundant information arising from the activity of large neuronal populations. Here we show that multichannel LFPs in monkey motor cortex each contain a slightly different mixture of distinctive slow potentials that accompany neuronal firing. As a result, the firing rates of individual neurons can be estimated with surprising accuracy. We implemented this method in a real-time biofeedback brain–machine interface, and found that monkeys could learn to modulate the activity of arbitrary neurons using feedback derived solely from LFPs. These findings provide a principled method for monitoring individual neurons without long-term recording of action potentials.

## Abstract (web summary)

The use of local field potential (LFP) brain signals may allow development of more efficient and robust neural prosthetic devices. Here, Hall *et al*. develop a method for estimation and biofeedback control of single-neuron firing rates using signals extracted from multiple low-frequency LFPs.

## Graphical abstract

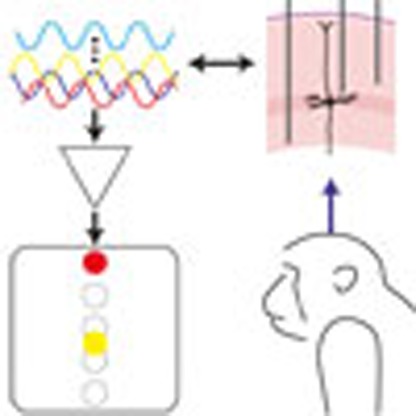

The ability to monitor the activity of neurons using electrodes implanted in the central nervous system has many potential applications in neuroprosthetic devices for patients with neurological injury and disease. For example, extracellular recordings of the spikes (action potentials) from multiple neurons in motor cortex can be used by brain–machine interfaces (BMIs) to control assistive devices^1^,^2^,^3^, or be relayed to other sites in the nervous system via electrical stimulation to provide artificial connections with therapeutic benefit^4^,^5^. However, the clinical translation of spike-based neuroprostheses faces two major challenges. First, micromotion of electrode implants and the tissue response around recording sites prohibit the recording of spikes from the same ensemble of neurons over prolonged periods^6^,^7^,^8^, reducing the stability and longevity of operation. Second, discriminating spike activity involves sampling, processing and/or transmission of signals at high rates (at least 10 kHz), which requires high power consumption and constrains implementation in implanted devices^9^.

Local field potentials (LFPs) offer an attractive solution to both of these problems. Since they reflect the summation of postsynaptic potentials over at least a few hundred micrometres from the recording site^10^,^11^,^12^,^13^,^14^,^15^, LFPs may be less sensitive to micro-movements and encapsulation of electrodes^16^,^17^, and kinematic information can be retrieved even from electrodes without clear spike activity^18^,^19^. Of particular interest is the low-frequency LFP (*lf*–LFP; below 5 Hz) because it can be sampled, processed and/or transmitted at rates comparable to the frequency content of movements. Historically, this ‘delta’ band has been associated with slow-wave sleep during which neurons become highly synchronized across wide areas of cortex and subcortical structures, but recent studies in awake subjects suggest that motor cortical *lf*–LFPs during upper-limb movement contain considerable information about kinematics^20^,^21^,^22^,^23^,^24^. Despite these advances, the physiological significance of the *lf*–LFP and its relationship to neuronal spiking are still poorly understood, hindering the applications of this signal beyond ‘biomimetic’ decoding of kinematics. For example, it is known that acquisition of BMI skill is often associated with profound changes in the tuning of neurons contributing to the decoder^1^,^2^,^25^. Although such changes can be reduced by recalibrating decoding algorithms online^26^, neuroplasticity may nevertheless be beneficial for the retention of BMI skill, as well as resistance to interference from other tasks^27^. These tuning changes likely reflect the ease with which the activity of individual neurons can be modulated under operant conditioning or biofeedback paradigms^28^,^29^,^30^. Previous studies have demonstrated that LFP power in broad frequency bands can be modulated under biofeedback^31^ and closed-loop BMI paradigms^32^. However, it is less clear which features of the *lf*–LFP can similarly be brought under volitional control and would thus be applicable for closed-loop BMIs.

We hypothesized that if we could identify *lf*–LFP components with a strong and consistent relationship to the firing rates of local neurons, then these should also be amenable to operant conditioning and therefore provide useful features for closed-loop BMIs. Moreover, the ability to infer firing rates from *lf*–LFP would be generally applicable for any neuroprosthetic application requiring long-term monitoring of neural activity. The correlation between single neurons and individual LFPs has been investigated extensively using spike-triggered averaging and more sophisticated techniques^15^,^33^,^34^,^35^,^36^,^37^. However, to date there have been surprisingly few studies of the extent to which neural firing rates can be estimated from multiple LFPs and vice versa, perhaps in part due to the persisting assumption that multiple LFPs convey largely redundant information arising from the synchronous activity of many neurons^10^,^22^,^38^. In the monkey visual cortex a single LFP channel has been estimated by linear summation of multiple potentials associated with spike activity^34^,^35^. Moreover, Rasch *et al*.^33^ found that in anesthetized (but not awake) animals, the firing rate of a single neuron could be predicted from a single channel of LFP. However, this study did not examine whether performance could be improved by using multiple LFPs. In the motor cortex, Bansal *et al*.^23^ have used multiple LFPs to decode the summed spiking activity of all neurons recorded on an electrode array, but did not investigate the possibility of predicting the firing rates of individual neurons.

We therefore examined the relationship between neural spiking and multichannel *lf*–LFPs recorded from the motor cortex of monkeys. We found that each *lf*–LFP could be well described by a linear mixture of spike-related slow potentials, and that the contribution of a given neuron to this mixture varied substantially across different *lf*–LFP channels. This variability, which was captured by a small number of distinct sources, allowed the estimation of firing rates of individual neurons from multichannel *lf*–LFPs with surprising accuracy. Estimates were stable over many days, captured a significant portion of the neural firing space and generalized well across abstract biofeedback tasks. Monkeys could increase or decrease the estimated activity of arbitrary neurons on demand, and even dissociate two cells using feedback based solely on *lf*–LFP features. Moreover, task-related firing rate modulations were specific to the targeted neurons. These findings reveal a wealth of information about local spiking activity contained within multiple *lf*–LFPs and could, in the future, allow low-power implantable neuroprostheses to infer neural activity long after spike recordings are lost.

## Results

### Estimating low-frequency LFPs from neuronal firing rates

**Figure 1:**
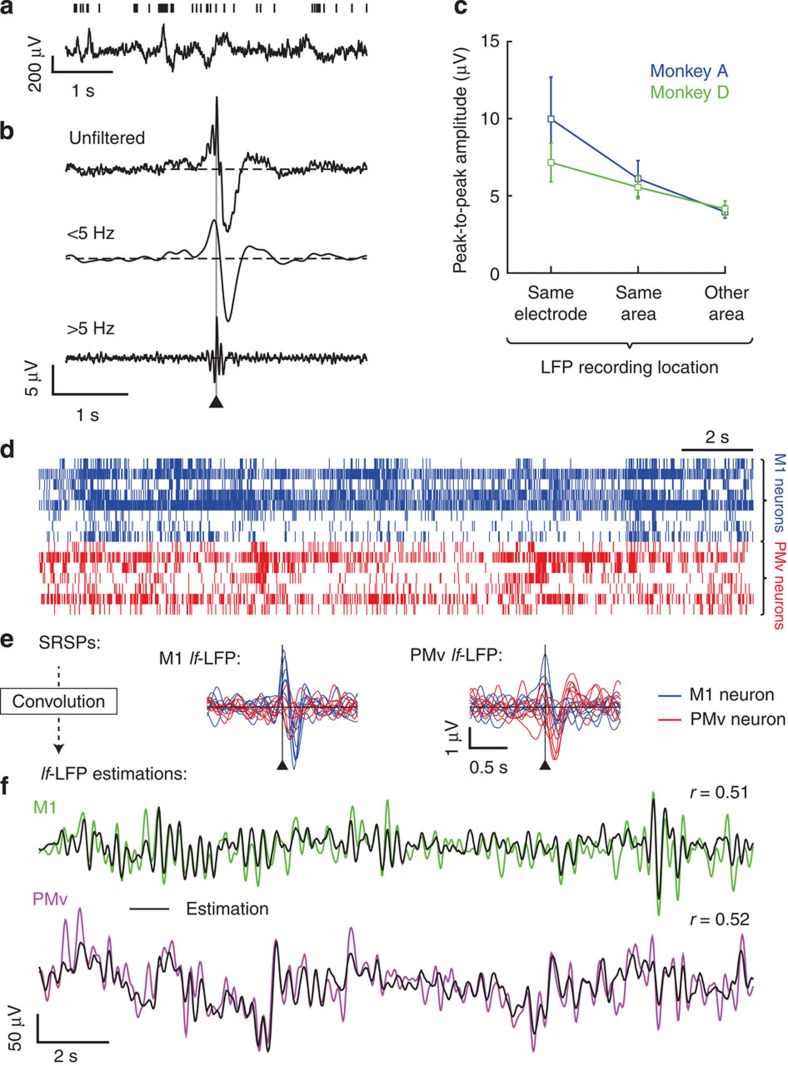
Estimating *lf*–LFPs from firing rates. (**a**) Spike raster of a single neuron (top) and LFP (bottom) recorded in M1. (**b**) STA of LFP (top), separated into low- (middle) and high-frequency (bottom) bands. (**c**) Mean peak-to-peak amplitude of STA of low-frequency LFP recorded on the same electrode as a neuron, other electrodes in the same cortical area (M1 or PMv) and electrodes in the other cortical area. Error bars show±s.e.m. (*n*=46 neurons per monkey). (**d**) Spike rasters for eight M1 (blue) and seven PMv neurons (red). (**e**) SRSPs for each neuron within a single M1 (left) or PMv *lf*–LFP (right). ▴ Indicates time of spike. (**f**) *lf*–LFPs estimated using a linear model applied to validation data. Significance thresholds (*P*<0.05, two-tailed; non-parametric bootstrap) for the indicated *r*-values were 0.07 and 0.11 for the M1 and PMv LFP, respectively. Data from monkey D.

**Figure 2:**
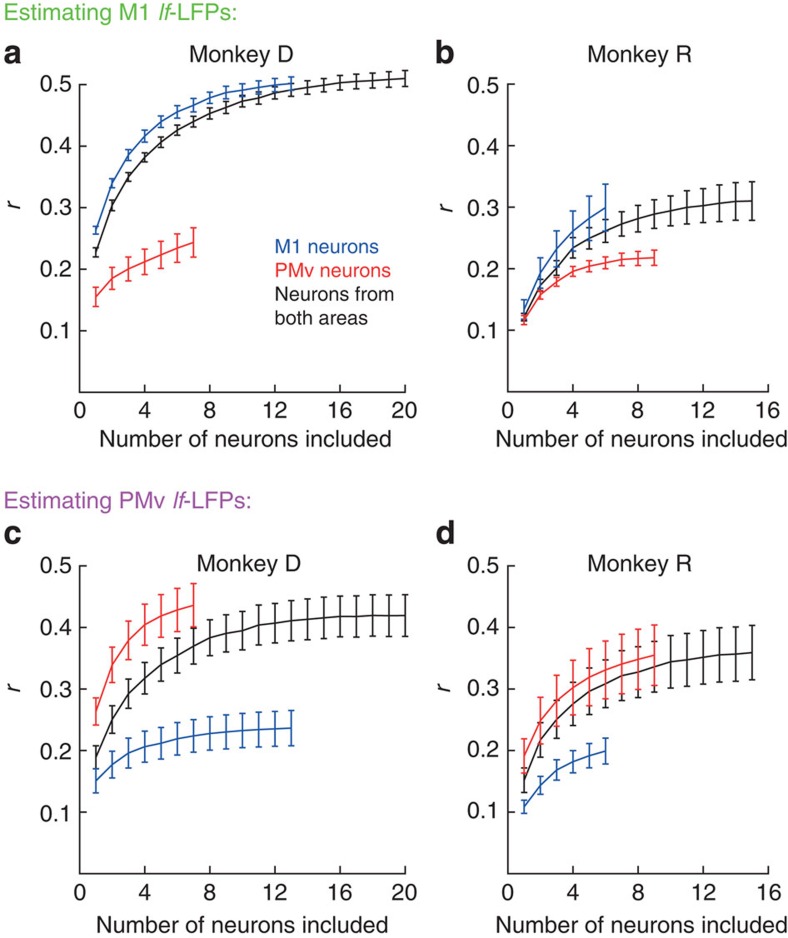
Neuron-dropping curves for *lf*-LFP estimation. (**a**) Average correlation coefficient over validation data for M1 *lf*–LFPs in monkey D, estimated using an increasing number of neurons in M1 (blue), PMv (red) or both areas combined (black). Error bars show±s.e.m. (*n*=11 *lf*–LFPs). (**b**) Same for M1 *lf*–LFPs in monkey R (*n*=10). (**c**) Same for PMv *lf*–LFPs in monkey D (*n*=11). (**d**) Same for PMv *lf*–LFPs in monkey R (*n*=12).

We recorded LFPs and spike activity (Fig. 1a) using moveable microwire arrays^39^ implanted in the right primary (M1) and ventral premotor (PMv) cortices of three monkeys performing a two-dimensional (2D) centre-out isometric wrist torque task using their left hand fixed in a static manipulandum (see Supplementary Fig. 1; Supplementary Methods). Spike-triggered averages (STAs) of LFP (Fig. 1b) typically exhibited beta-band (~20 Hz) phase-locking, as well as large and consistent low-frequency features, which we refer to as spike-related slow-potentials (SRSPs). The SRSP amplitude was typically largest in the LFP recorded from the same electrode as the spikes used to construct the average (Fig. 1c). However, robust SRSPs were also observed in recordings from other electrodes within the same cortical area, suggesting the LFP contains a mixture of slow components reflecting the activity of both neighbouring and distant neural activity within each cortical area. To test this, the low-frequency LFP (*lf–*LFP; filtered<5 Hz) was modelled as a sum of the spike trains of multiple neurons (Fig. 1d) convolved with filter kernels that resembled the SRSP waveforms (Fig. 1e, see Methods). When tested on validation data that were not used to build the model, we were reliably able to estimate *lf–*LFPs recorded from both M1 and PMv (Fig. 1f). The quality of fit increased monotonically as more neurons were included, with the majority of useful information obtained from those recorded within the same cortical area as the estimated *lf*–LFP (Fig. 2). The local nature of the SRSP was confirmed by its polarity inversion as electrodes were progressively advanced through the cortical grey matter (Supplementary Fig. 2).

### Estimating single-neuron firing rates from multiple *lf*–LFPs

**Figure 3:**
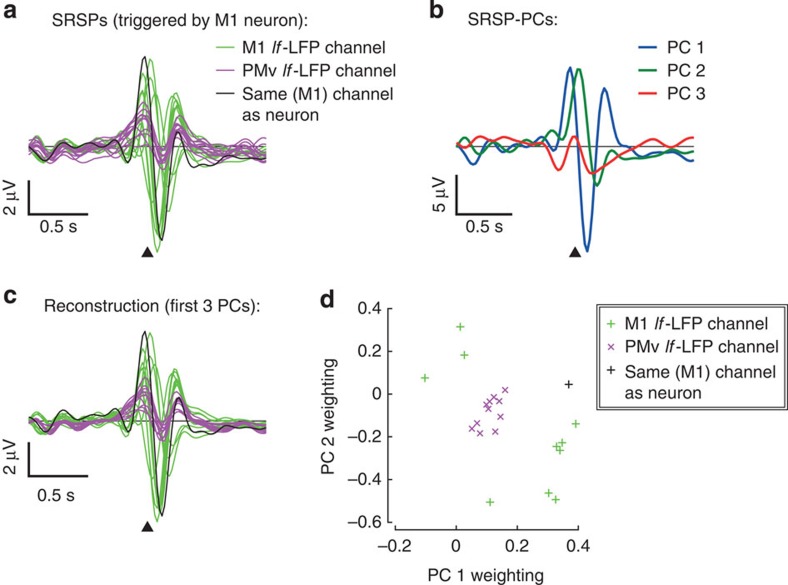
PC decomposition of the SRSP. (**a**) SRSPs of a single M1 neuron with multiple *lf*–LFPs from M1 (green) and PMv (purple) in monkey D. Black trace shows the *lf*–LFP recorded on the same channel as the trigger neuron. ▴ indicates time of spike. (**b**) First three principal components (PCs) of the SRSPs. (**c**) Reconstruction of the SRSPs in **a** using only the first three PCs. (**d**) Scatter plot showing the weightings of PC 1 and PC 2 used for this reconstruction.

**Figure 4:**
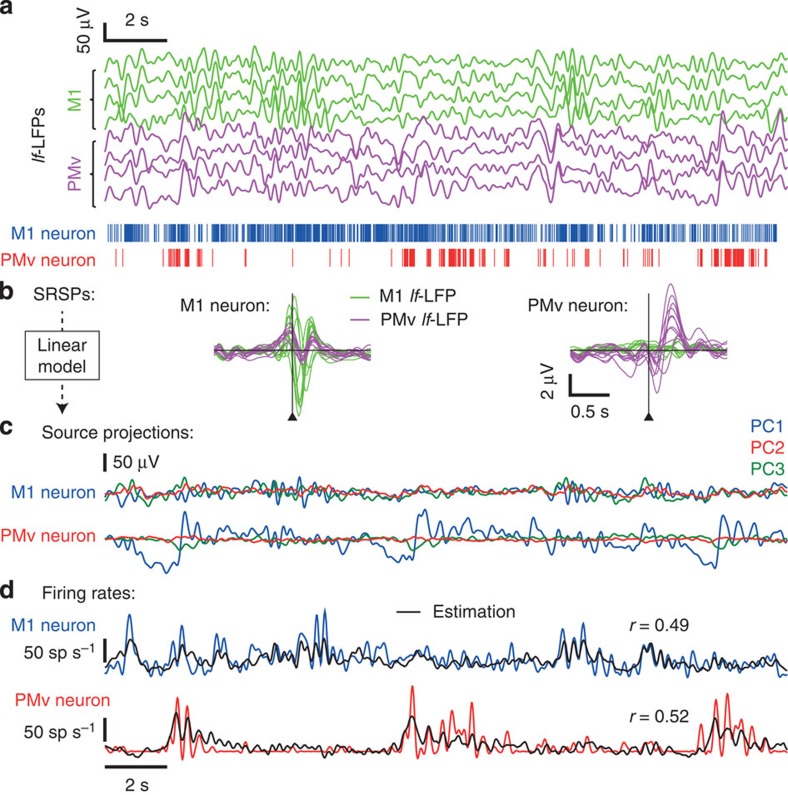
Estimating firing rates from *lf*–LFPs. (**a**) Example *lf*–LFPs (top) and spike rasters (bottom) recorded from monkey D. (**b**) SRSPs across 20 *lf–*LFPs associated with a single M1 (left) and PMv (right) neuron. (**c**) Source projections for each neuron, representing the *lf–*LFP mixture that best estimates the contribution of each SRSP-PC. (**d**) Firing rates estimated by deconvolution of source projections. Significance thresholds (*P*<0.05, two-tailed; non-parametric bootstrap) for the indicated *r*-values were 0.11 and 0.10 for the M1 and PMv neuron, respectively.

Importantly, the SRSP associated with an individual neuron varied in both shape and polarity across different *lf*–LFP electrodes, particularly within the same cortical area as the trigger neuron (Fig. 3a), despite the fact that *lf*–LFPs themselves appeared broadly similar (Fig. 4a). However, this variation could be explained by only a few principal components (PCs) (Fig. 3b–d), suggesting that the contribution of a particular neuron to the multichannel *lf*–LFP comprises a limited number sources with distinct spatio-temporal profiles. On the basis of this, we developed a method to estimate these sources from the *lf*–LFP, and then used deconvolution to recover the firing rate of single neurons (see Methods and Supplementary Fig. 3).

**Figure 5:**
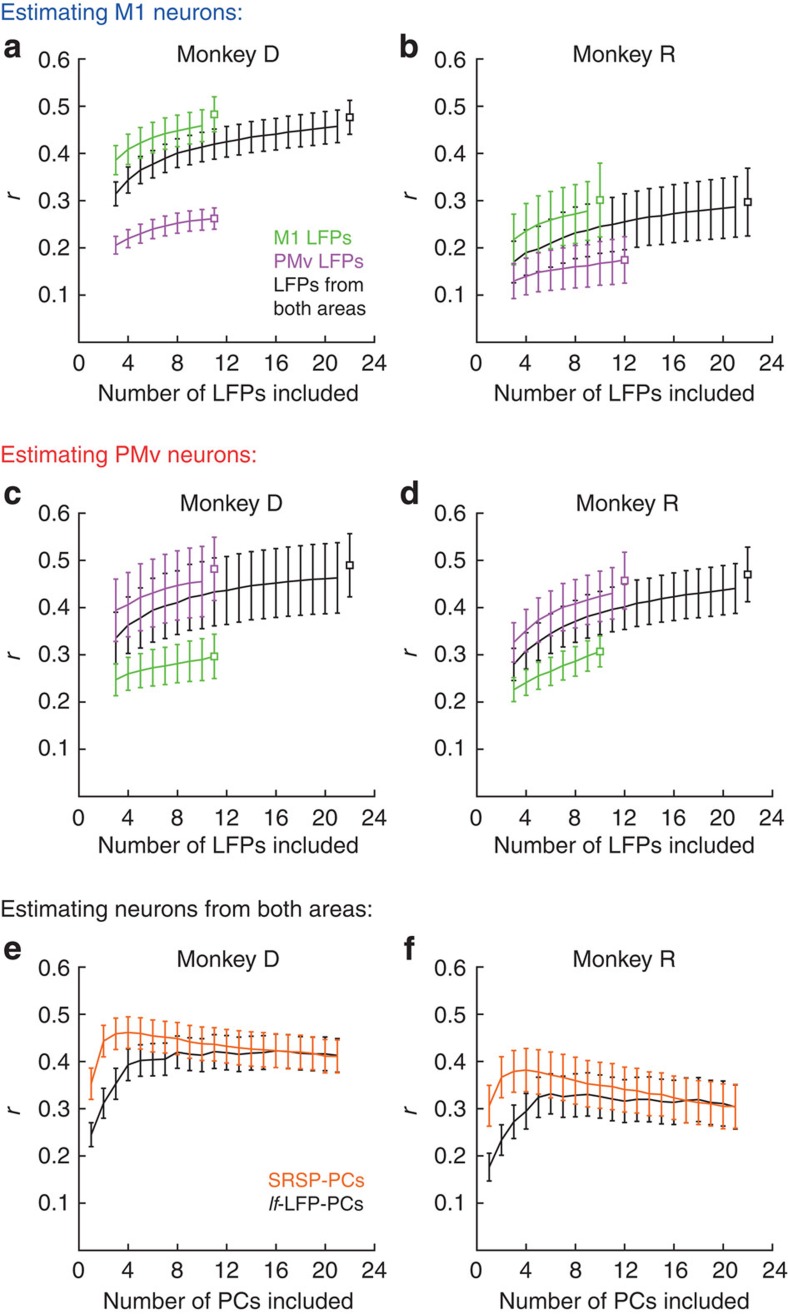
LFP- and PC-dropping curves for firing rate estimation. (**a**) Average correlation coefficient over validation data for M1 neuron firing rates in monkey D, estimated from increasing numbers of *lf*–LFPs from M1 (green), PMv (purple) or both areas combined (black). Dimensionality reduction was performed using three SRSP-PCs. Open squares show the result when the *lf*–LFP on the same electrode as the estimated neuron was also included. Error bars show±s.e.m. (*n*=13 neurons). (**b**) Same for M1 neurons in monkey R (*n*=6). (**c**) Same for PMv neurons in monkey D (*n*=7). (**d**) Same for PMv neurons in monkey R (*n*=9). (**e**) Performance when estimating firing rates of all neurons in monkey D using increasing numbers of SRSP-PCs (orange), compared with a model based on PCs of *lf*–LFPs (black). All 22 LFPs were used (but with exclusion of the *lf*–LFP on the same electrode as each estimated neuron). Error bars show±s.e.m. (*n*=20 neurons). (**f**) Same for all neurons in monkey R (*n*=15).

First, we decomposed the SRSPs for a particular neuron (Fig. 4b) into PCs and convolved each with the neuron’s firing rate to estimate its contribution to the *lf*–LFP. We found the low-dimensional projection of the *lf*–LFP data that best approximated the first few source estimates (Fig. 4c), and used Wiener deconvolution to recover the firing rate. To exclude the unlikely possibility of action potential waveforms passing our low-pass filter^40^, the *lf*–LFP recorded from the same electrode as the neuron was not used for its firing rate estimation. Once the projection matrix and deconvolution filter had been obtained from training data, their ability to estimate firing rates was tested on separate validation data. Figure 4d shows a representative validation performance using 20 LFPs to estimate the firing rate of single neurons in M1 and PMv. Firing rate estimates captured a significant proportion of modulation across the low-frequency band (Supplementary Fig. 4a,b). Moreover, the task-related modulation of individual neurons during the torque task, including trial-averaged firing rate profile and ‘preferred direction’ could be retrieved from LFP-based firing rate estimates (Supplementary Fig. 4c,d). The quality of firing rate estimation increased with the number of *lf*–LFPs included in the model, with the most useful being within the same cortical area as the estimated neuron (Fig. 5a–d). Firing rate estimation was only marginally improved by further inclusion of the *lf*–LFP recorded on the same electrode as the estimated neuron (Fig. 5a–d, open squares).

Our approach to recovering firing rates from low-dimensional source estimates was motivated by the observation that the contribution of a single neuron to the *lf*–LFP (that is, the SRSP) comprised mixtures of a small number of components, but these will not necessarily be the largest signals within the *lf*–LFP. As a result, estimation based on SRSP components out-performed models fitted directly to the PCs of the *lf*–LFPs (Fig. 5e,f). Moreover, validation performance was optimal when only a limited number of sources were included, suggesting that each SRSP contains about three to four distinct components.

### LFP-based firing rate estimates are stable over many weeks

**Figure 6:**
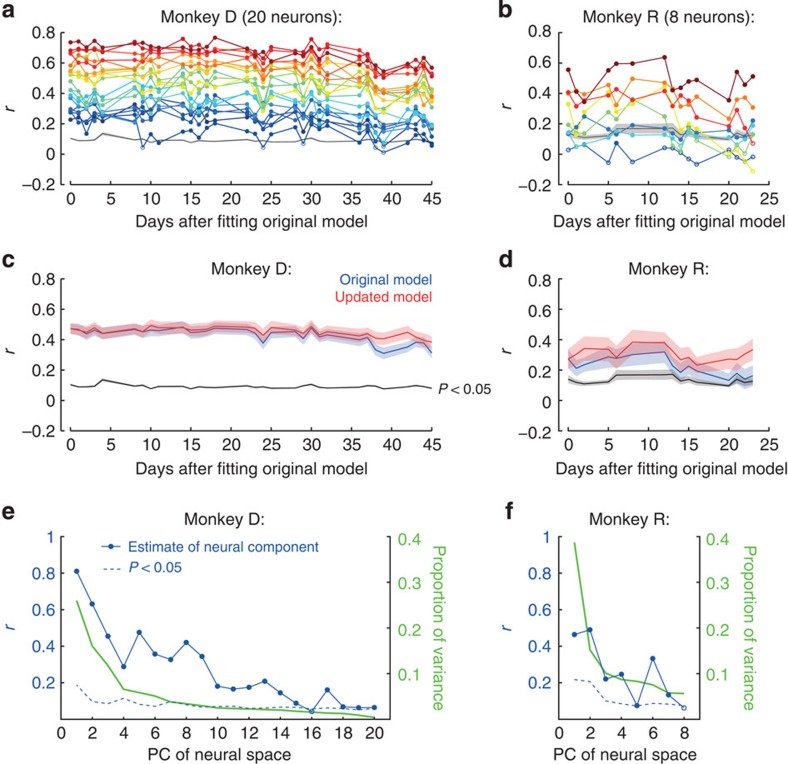
Long-term stability and dimensionality of firing rate estimation. (**a**) Estimation of 20 neurons’ firing rates, using 22 *lf*–LFPs in monkey D over 45 days, without model updating. Filled circles indicate significant estimation (*P*<0.05, two-tailed; non-parametric bootstrap). Neurons are colour-coded according to quality of estimation on day 0. Black line shows an illustrative significance threshold (mean threshold of *n*=20 neurons; shading shows±s.e.m), although each firing rate undergoes its own significance test. (**b**) Same for monkey R; 8 neurons, using 22 *lf*–LFPs, over 23 days. (**c**,**d**) Performance (mean correlation coefficient) of the original model (blue, fitted to data on day 0) versus an updated model (red, fitted to the data daily). Shading shows ±s.e.m. (monkey D, *n*=20 neurons; monkey R, *n*=8 neurons). (**e**,**f**) Proportion of variance in actual firing rates of all neurons captured by each PC (green), and correlation coefficient on validation data for the projection of estimated firing rates onto the PCs of actual firing rates (blue). Dashed line indicates *P*<0.05 significance threshold (two-tailed; non-parametric bootstrap).

To assess the stability of the relationship between *lf*–LFPs and firing rates, we recorded the same ensemble of neurons over a prolonged period (monkey D: 20 neurons, 45 days; monkey R: 8 neurons, 23 days). Models fitted to data recorded on day 0 were able to estimate firing rates of most neurons simultaneously from validation *lf*–LFP data recorded during that session (*r*-values for monkey D: mean 0.47, range 0.23–0.74; monkey R: mean 0.27, range 0.03–0.56). We then used the same model parameters to predict cell activity on subsequent days (Fig. 6a,b. *r*-values on the last day for monkey D: mean 0.31, range 0.06–0.57; monkey R: mean 0.16, range −0.11 to 0.51). Performance of the model using parameters from day 0 was stable throughout the recording period and was only slightly improved by fitting new parameters on each day (Fig. 6c,d; see Methods).

Next, we examined how well the trial-averaged modulation of single-neuron firing rates (aligned to the end of the successful hold period) could be retrieved from LFP-based estimates. On day 0, the mean (±s.d.) correlation between the average firing rate of single neurons and the equivalent average of the LFP-based estimates was *r*=0.93±0.06 for monkey D and 0.88±0.14 for monkey R (Supplementary Figs 5a and 6a). Note that these correlation coefficients are considerably higher than the *r*-values obtained for non-averaged data, suggesting that our method gave an unbiased estimate of task-related modulations of single neurons that converged towards the same trial-averaged profiles as the instantaneous firing rates. On subsequent days, despite slight variation in the animals’ behaviour, trial-averages of LFP-based estimates continued to resemble the actual trial-averaged firing rates from day 0 (*r*-values on day 1: 0.86±0.11 for monkey D and 0.59±0.26 for monkey R; *r*-values on the last day: 0.74±0.11 and 0.53±0.27). Remarkably, trial-averaged LFP-based estimates were as similar to the day-0 profiles as were trial-averages of the actual firing rates on subsequent days (day 1: 0.81±0.13 for monkey D and 0.63±0.32 for monkey R; last day: 0.71±0.15 and 0.44±0.32; Supplementary Figs 5b and 6b). Comparable results were obtained when we compiled trial-averaged profiles for each target direction separately, albeit with lower *r*-values for both actual and estimated firing rates as a consequence of averaging over fewer trials (Supplementary Figs 5c and 6c). Therefore, if we take as ‘ground truth’ the task-related modulation of a single neuron on day 0, this could be recovered from the LFP-based firing rate estimates at least as accurately as from the actual firing rate of the same neuron across the extended recording period in both animals.

We were still able to recover trial-averaged profiles from the LFP-based estimates even for the last sessions in our data sets before electrodes were moved to find new neurons, obtaining *r*-values of 0.66±0.14 for monkey D and 0.57±0.34 for monkey R at time points corresponding to days 116 and 63, respectively. It is not possible to provide comparable values for the actual firing rates in these sessions since spike waveforms recorded on the electrodes had changed and/or deteriorated to the extent that the original ensemble of neurons could no longer be identified.

### Dimensionality of LFP-based firing rate estimates

In general, the firing rates of neurons within the same cortical area exhibited higher correlation than across areas, although we did not find clear evidence for smaller ensembles of tightly correlated cells within each area (Supplementary Fig. 7a–f). Nevertheless, we considered the possibility that multichannel *lf*–LFPs might contain information only about a limited number of hidden or latent variables, corresponding to correlated components of the population activity. In this case, LFP-based firing rate estimates would predict only a few dimensions of the observed neural space. To test this, we calculated the PCs of the actual firing rates of all recorded neurons (low-pass filtered at 5 Hz) so as to find dimensions of the neural space that captured the greatest co-variation (Fig. 6e,f, green lines). Approximately half of the total firing rate variance in both animals was explained by the first two PCs (which generally captured broad co-activation of neurons within M1 or PMv; Supplementary Fig. 7g–i), with the remainder distributed across the higher components. We then projected the LFP-based firing rate estimates of all neurons onto the same PC axes, and assessed their correlation with actual firing rates along each dimension using validation data (Fig. 6e,f, blue lines). The highest correlations were obtained for the first two PCs, consistent with a previous observation that the total spiking within an area can be decoded from *lf*–LFPs^23^. However, we could also obtain statistically significant prediction of all but one of the higher PCs in each animal, suggesting fractionated components of the population activity were also contained within the LFP-based estimates. This was despite the fact that higher PCs captured less of the firing rate variability and would therefore be expected to have a lower signal-to-noise ratio. Indeed, the drop in estimation performance with increasing PC number could be simulated by artificially introducing noise uniformly across the actual spike recordings. A proportion of spikes from each neuron were shuffled to spike trains of other neurons in the same recording (in effect simulating what might happen in a real experiment if spikes were misclassified to the wrong cell; see Supplementary Methods). Performance of the LFP-based firing rate estimate over the entire neural space was comparable to firing rates calculated from actual spike data, in which 25% of spikes were correctly classified (Supplementary Fig. 7j–l). This is consistent with the mean *r*-values for single-neuron estimation (~0.5), suggesting on average 25% of the true firing rate variation of individual neurons was captured by LFP-based estimates. Note, however, that while we here simulated the effect of noise by adding spikes from other neurons, the actual noise in our firing rate estimates did not have any consistent task relationship, since trial-averaged profiles converged on the true task-modulation of the single neurons (Supplementary Fig. 4c,d).

### Generalization of LFP-based estimates across BMI tasks

Next we asked whether the relationship between LFPs and firing rates would generalize across behaviours, or vary systematically with different firing rates and altered neuronal correlations. Therefore we explored how well models built on data recorded during the isometric torque task could be used to estimate firing rates during various biofeedback BMI tasks (see Supplementary Fig. 1). The position of a one-dimensional (1D) cursor was controlled in real-time by either the firing rate of one arbitrarily chosen cell, the summed firing rates of two cells or the difference in firing rates of two cells (see Supplementary Methods). Models of both cells’ firing rates built on torque task data generalized well across all three conditions, performing only marginally worse than individual models built and validated on data within each condition (Supplementary Fig. 8a,b; see also Supplementary Discussion). During the one-cell control task, we quantified the degree to which modulation of neuronal firing rate was associated with movement of the wrist, using a ‘torque modulation index’ (see Supplementary Methods). We found a weak correlation (significant in one animal) between this index and the performance of our LFP-based firing rate estimate (monkey D, correlation coefficient=0.43, *P*=0.04, monkey A, correlation coefficient=0.30, *P*=0.16). However, LFP-based firing rate estimation performed well in many cases even when the behaviour involved minimal torque modulation (Supplementary Fig. 8e,f). In addition, the size and shape of the SRSP was conserved across trials requiring both increases and decreases in neuronal firing rates, including those not associated with overt wrist movement (Supplementary Fig. 9). Interestingly, consistent features in the trial-averaged *lf*–LFP could not alone account for the SRSP, suggesting that it reflected largely correlated trial-to-trial variability in the neuronal firing rate and LFP (Supplementary Fig. 9; see also Supplementary Discussion).

### Real-time biofeedback using LFP-based firing rate estimates

**Figure 7:**
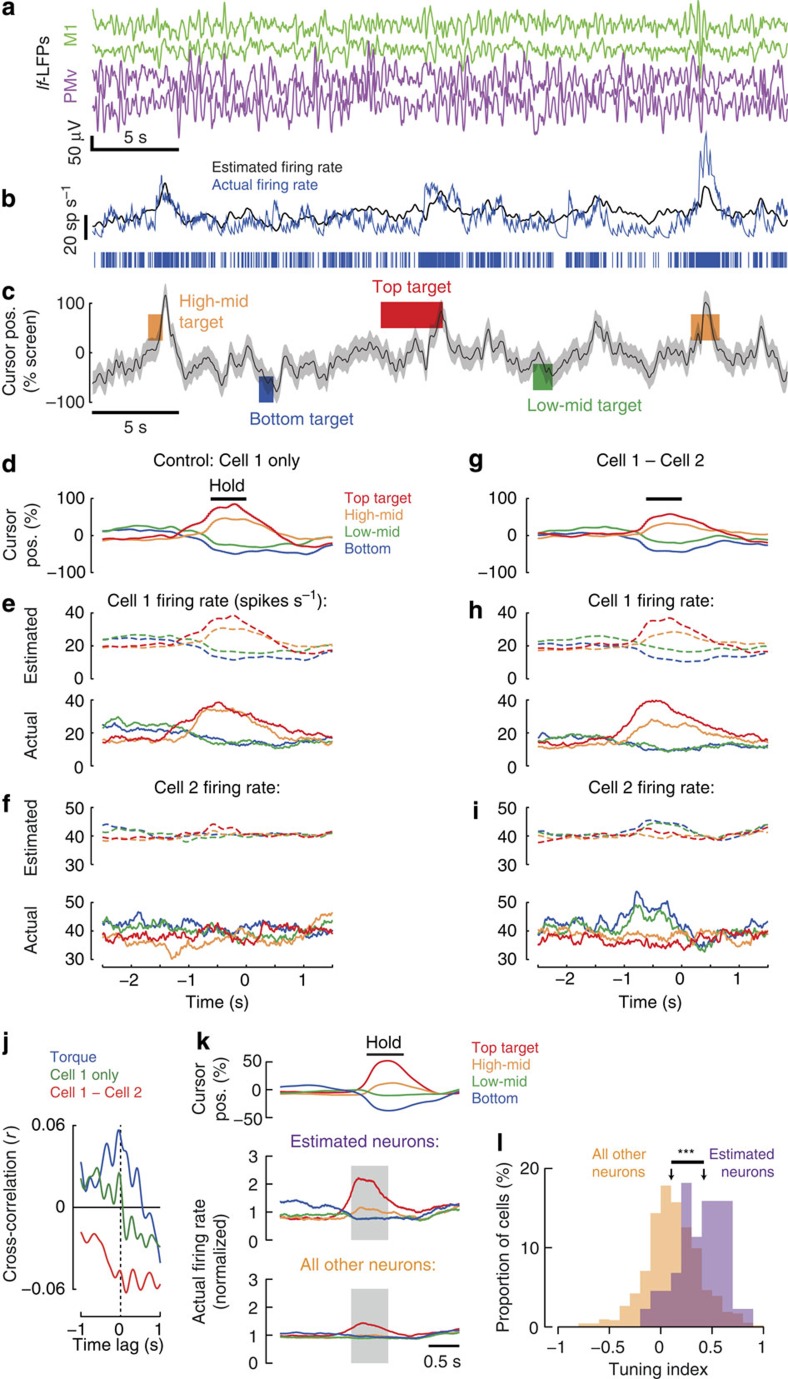
Biofeedback BMI controlled by real-time LFP-based firing rate estimates. (**a**) Example *lf*–LFPs (4 shown of 18) as monkey D controlled a BMI cursor using the estimated firing rate of an M1 neuron. (**b**) Real-time estimated and actual firing rates and spike raster. (**c**) Cursor and target positions (shading indicates cursor and target width). (**d**) Trial-averaged cursor position aligned to end of the successful hold period. (**e**) Trial-averaged estimated and actual firing rates of Cell 1 (the controlling M1 neuron). (**f**) Trial-averaged estimated and actual firing rates of Cell 2 (a non-controlling M1 neuron). (**g**–**i**) As in **d**–**f** now for a task controlled by the difference between estimated firing rates of Cell 1 and Cell 2. (**j**) Cross-correlation of the actual firing rates of Cell 1 and Cell 2 during the initial torque task and subsequent ‘LFP-control’ BMI tasks. (**k**) Mean cursor position (top) across sessions where monkey D (22 sessions) or R (22 sessions) controlled the estimated firing rate of one neuron. Mean actual firing rates of controlling neurons (middle, *n*=44) and all other cells (bottom, *n*=947). (**l**) Tuning index (calculated within grey box in **k**) of the estimated neuron versus all other cells. ↓, median; ***P<0.001, Mann–Whitney test.

Finally, to demonstrate the utility of LFP-based firing rate estimation for closed-loop control, we implemented our algorithm in real-time using a 2-s history of *lf*–LFP data to predict the instantaneous firing rates of two neurons 0.2 s in the past. Again, we excluded *lf–*LFPs from those electrodes used to record the selected neurons. After fitting the model on ~5 min of torque-tracking data, monkeys performed the 1D biofeedback BMI task now controlled by *estimated* firing rates of one or two neurons (Fig. 7a,b). Monkeys were quickly able to increase and decrease the estimated firing rates of single neurons (Fig. 7c–f), and achieve independent control of two estimates when each moved the cursor in opposite directions (Fig. 7g–i). Although we imposed no direct constraints on the activity of the underlying neurons, monkeys nevertheless performed the task by modulating the actual firing rates of the chosen neurons (Fig. 7d–i), and the correlation between neurons changed in accordance with the imposed biofeedback contingency (Fig. 7j). We defined a tuning index (see Methods) to quantify the modulation of firing rate with target position, and across 44 sessions in two animals the estimated neurons were tuned significantly more than the other recorded neurons (median tuning index of estimated neurons 0.42; other neurons 0.10; *U*=9,140, *P*<0.001, two-tailed, by the Mann–Whitney *U*-test) (Fig. 7k,l).

## Discussion

We have demonstrated that single-neuron firing rates can be estimated from multichannel *lf*–LFP recordings in motor cortex using simple linear models. LFP-based estimates capture around 25% of the variance of instantaneous firing rates and 75–85% of trial-averaged profiles, performing as well as the actual firing rates at reconstructing the task relationship of individual neurons on subsequent days. These models were remarkably robust (up to several months in both animals), generalized across behaviours and yet were sufficiently accurate and specific to permit operant conditioning of individual neuron firing rates using biofeedback based solely on *lf–*LFPs.

Our finding that the *lf*–LFP contains contributions from a number of sources reflecting the activity of local neuronal populations is in agreement with recent studies^33^,^35^,^41^. However, to our knowledge, this is the first demonstration that the different mixtures contained within multiple *lf*–LFPs can be separated for estimation and biofeedback control of single neurons. Given that LFPs are population signals, the accuracy with which we are able to resolve individual neurons is surprising, especially since neural synchrony inevitably confounds the inference of causal effects from STAs. Our use of linear models incorporating multiple recorded cells can only partially mitigate this problem. Clearly, given its large amplitude and slow time course, the SRSP attributed by the model to each recorded cell must contain contributions from unrecorded (but correlated) neurons in the local network. On one hand, since firing rate estimates generalized well across a variety of abstract BMI tasks we conclude that the contribution of unrecorded neurons remained broadly consistent across behaviours, although further experiments would be required to demonstrate the stability of SRSP features across more complex tasks. On the other hand, independent fluctuations in the firing rates among the recorded neurons are vital for their estimation since the contribution of perfectly correlated cells to the LFP would be impossible to separate. More plausibly, moderate correlations might be signatures of larger, synchronous ensembles generating the observed LFP. In this case, we would expect to predict only those correlated components of neural activity (reflecting the activity of these ‘hidden’ ensembles) and not uncorrelated dimensions (reflecting neural noise). However, when estimating the firing rates of 8–20 neurons simultaneously, we obtained significant prediction along all but one PC of the observed neural space. The quality of fit along each dimension could be simulated by adding noise uniformly across individual neurons, suggesting that the LFP-based estimates are not systematically biased to particular PCs. Moreover, when estimates of single neurons were used for real-time biofeedback, task-related firing rate modulations were largely confined to the targeted neuron. Therefore, we conclude that the dimensionality of LFP-based estimates is comparable to the dimensionality of our multiple single-unit recordings. It remains to be seen whether this will stay true as the density of recording arrays increases. One possibility is that, as more of the network is sampled, the SRSP attributed to each recorded neuron will eventually converge on the true causal effect of its spiking. Alternatively, greater single-unit resolution may ultimately reveal tightly correlated ensembles beyond which SRSP-based separation becomes impossible.

The origin and spatial extent of the LFP remains a subject to debate, with estimates ranging from a few hundred micrometres^11^,^12^ to several millimetres^13^. We observed robust SRSPs varying in shape and polarity within *lf*–LFPs recorded on different electrodes throughout the same cortical area as the trigger neuron. We can rule out recording artefacts (for example, room noise, head movements and electromyogram) since the SRSP reversed polarity within the grey matter and *lf–*LFPs were most informative of the firing rates of neurons within the same cortical area. Moreover, when controlling LFP-based firing rate estimates, monkeys selectively modulated the firing rates of the estimated neurons rather than performing behaviours that might be expected to generate artefacts. Although we cannot discount the presence in our LFP recordings of artefacts uncorrelated with spiking activity, these would only reduce the accuracy with which we could estimate firing rates. We cannot say whether the spatial extent of the SRSP reflects volume conduction of local sources or synchronization of broader neuronal populations within each cortical area^42^. Nevertheless, LFP-based firing rate estimation worked equally well for M1 and PMv, while similarities with spike–LFP relationships reported in macaque visual cortex^33^,^34^ and rat somatosensory and prefrontal cortex^36^ suggest that the SRSP may reflect a ubiquitous feature of cortical organization rather than a unique property of motor cortex. Possible mechanisms that could account for low-frequency SRSP components occurring several hundred milliseconds after spike activity include GABA_B_-mediated recurrent inhibition^43^,^44^—since the slow kinetics of the G-protein-coupled receptor gives rise to extracellular potentials that can be delayed substantially relative to cell activity^45^—as well as intrinsic effects, such as slow hyperpolarization-activated *I*_h_ currents that contribute to low-frequency resonances in cortical neurons^46^. Alternatively, reciprocal connections with the thalamus form feedback loops that are thought to contribute to delta-frequency oscillations^47^. Indeed, our finding that the SRSP contains 3–4 distinct components suggests that multiple processes likely contribute. Because different electrodes record different mixtures of these components, we are able to extract information about firing rates that may not be present in global measures such as overall LFP power in broad frequency bands. Moreover, information in LFPs is highly layer dependent^41^,^48^ and variation in electrode depth within our recording array appears beneficial for extracting non-redundant spike-related features from multiple LFP channels. This may additionally help explain why decoding of LFPs typically outperforms surface recordings from the brain or scalp^18^,^20^.

Large regression models can suffer from over-fitting and instability, especially when inputs are highly correlated^49^ (as in the case of multiple LFPs^38^). However, the distinct SRSP components associated with each neuron allowed us to develop a biophysically principled approach to dimensionality reduction, which improved model validation and out-performed PC regression. Our assumption of a linear relationship between *lf*–LFP and spike activity is likely suboptimal, although evidence from the primary visual cortex suggests that nonlinear approaches may yield only marginal improvements^33^. Experiments in the anesthetized visual cortex suggest there may be distinct information contained in the *lf*–LFP compared with gamma-band LFPs and spikes^50^, and that inclusion of these higher frequencies improves prediction of spiking^33^. LFP spectra in the awake motor cortex are characterized by strong activity in the beta-band, but this is typically suppressed during movement while spiking activity shows the greatest modulation. This motivated our use of the low-frequency band for firing rate estimation. Nevertheless, it is possible that the inclusion of higher frequency bands could further improve performance, albeit at the cost of increased computational complexity.

The instability of single-unit recordings is a challenge for invasive neuroprostheses, but the wide spatial extent of the SRSP across electrodes within the same cortical area suggests the *lf*–LFP may be less sensitive to micromotion than extracellular spike recordings. This may explain why we could still recover trial-averaged firing rate profiles after several months. Remarkably, this means that even after a neuron on one electrode is lost (or the entire electrode signal is lost), its firing rate can still be inferred from the *lf*–LFP recorded on other electrodes. We propose that soon after electrode implantation, while clean spike recordings can be obtained from many neurons, model parameters that relate the firing rates of these neurons to *lf*–LFPs should be calculated. Such an approach may allow firing rate estimation also to be performed after spike recordings have substantially deteriorated. While recent studies suggest that unsorted threshold crossings can be used for biomimetic BMIs over several years^17^,^51^, it remains to be seen whether the day-to-day stability of these signals over extended periods will match LFPs^17^,^18^,^19^. Moreover, *lf*–LFPs can be sampled at much lower frequencies, which has important implications for the power requirements of implantable devices. Once model parameters have been determined, real-time firing rate estimation requires only two simple steps: projection of the multichannel *lf–*LFP data into a low-dimensional source space followed by linear deconvolution. Such processing would be relatively easy to implement using low-power electronics, particularly as it can operate at sampling rates as low as tens of Hertz. The ability to estimate firing rates from *lf*–LFP extends the use of this signal beyond biomimetic decoding from open-loop training data, an approach which has limitations for clinical application in paralysed subjects. The true potential of BMIs as abstract tools that are operated skilfully by the user will likely be achieved through extensive ‘closed-loop neural adaptation’^25^,^27^,^29^ at the level of individual neurons or small ensembles. Indeed, estimation of firing rates from *lf*–LFPs could in principle be used within any device requiring monitoring of neuronal activity over extended periods of time, with encouraging implications for the development of robust and efficient neuroprostheses for a range of neurological conditions.

## Methods

### Subjects and training

Three female monkeys (*Macaca mullata*) were used in this study: monkey A (age at the start of the experiment 5 years, 9 months; weight 9.7 kg), monkey D (5 years, 4 months; 6.4 kg) and monkey R (5 years, 4 months; 5.4 kg). Subjects were trained to sit in a primate chair, and voluntarily accept neck and arm restraint, and immobilization of the left hand within a static manipulandum, but were not head-fixed. All animal procedures were carried out under appropriate UK Home Office licenses in accordance with the Animals (Scientific Procedures) Act 1986, and were approved by the Local Research Ethics Committee of Newcastle University.

### Wrist torque-controlled task

We trained subjects to perform a task in which 2D isometric left-wrist torque (measured by a static six-axis force/torque manipulandum) controlled the 2D position of a circular cursor on a screen placed ~50 cm in front of the subject. We refer to this here as the ‘torque task’. Each trial was initiated by the cursor entering a central circular ‘home’ region, reflecting zero torque (relaxation). A peripheral circular target appeared at one of eight positions spaced equally around a circumference centred on the home position. After a variable ‘cue’ period (between 1.2 and 2.4 s)—during which subjects had to remain in the home region—they were required to move the cursor to overlap the target for a fixed ‘hold’ period (0.6 s). If successful, subjects heard a reward tone, and were given a small piece of fruit reward by a researcher. There was no time limit for an individual trial. Around 300–500 trials were performed per day in a single session.

### Brain-machine interface tasks

Following surgical implantation, subjects also performed one of two different types of biofeedback BMI tasks, in which the 1D screen cursor position was controlled by the normalized amplitude of signals derived from neural recordings, to acquire 1D targets. Each day’s recording consisted of 50 trials of the torque task followed by 250–450 trials of a BMI task. In general, for BMI tasks we tried to choose neurons with large amplitude, clean spikes, but did not otherwise select based on task-related modulation of firing rate. Moreover, the axis of 1D cursor movement under brain control was chosen at random so as to have no consistent relationship with the preferred direction of neurons. There were two main types of BMI tasks: ‘cell control’ and ‘LFP control’. Monkey A performed only the ‘cell-control’ task. Monkey D first performed a series of ‘cell-control’ sessions followed, after an intervening period of 6 months, by the ‘LFP-control’ task, based on a different sample of neurons. Monkey R performed only the ‘LFP-control’ task. The ‘cell-control’ BMI task is described in Supplementary Methods.

### ‘LFP-control’ BMI task

In this task, the 1D cursor position was controlled by the smoothed estimated firing rates of neurons. Each day, we built a model using data from the torque task that was then used to estimate simultaneously the firing rates of two neurons from *lf*–LFP data in real-time. The two estimated firing rates (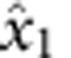 and 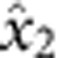) were smoothed online using an exponential decay filter with a decay constant *α*=250 ms. Cursor position was then controlled by either the smoothed estimated firing rate of one neuron or by the difference between two smoothed firing rate estimates. Because the firing rates of different units were modulated over different ranges, we applied a linear scaling to normalize rate estimates to screen co-ordinates, based on the distribution of the firing rate estimates obtained during the torque task. Each firing rate estimate was mapped to normalized screen co-ordinates such that the 5th/95th centiles of this distribution corresponded to ±50% (in screen coordinates, where 100% represents the screen edge). Targets appeared at four positions: ±35 and ±70%. During one-cell control, the cursor position, *c*, was equal to the scaled firing rate, 
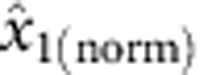
. During two-cell control, 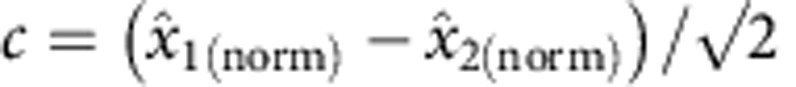, where the factor of 
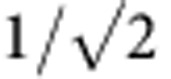
 was used to make all targets equidistant from the origin in the 2D normalized neural space.

### Surgical procedures

Surgeries were performed under general anaesthesia induced with propofol (2-4 mg kg^−1^) and maintained with sevofluorane (minimum alveolar concentration 1.8-1.9%) and alfentanil infusion (0.2 μg kg^−1^ min^−1^). Ventilation was supported and expired carbon dioxide concentration and peripheral oxy-/deoxy-haemoglobin ratio were monitored. An intra-arterial line and urethral catheter were inserted to monitor circulatory status, and intravenous fluid support was provided (Hartmann’s saline, 5–10 ml kg^−1^ h^−1^), while body temperature was maintained between 36.5 and 37.5 °C throughout. Animals received peri-operative methylprednisolone (5.4 mg kg^−1^ h^−1^) and cefotaxime (250 mg, 2-hourly), as well as post-operative antibiotics (ceftiofur 3 mg kg^−1^), analgesia (meloxicam 0.2 mg kg^−1^) and steroids (methylprednisolone).

Guided by a prior structural magnetic resonance imaging scan, we made individual craniotomies over the primary motor cortex (M1; coordinates antero-posterior [AP] ~11 mm, medio-lateral [ML] ~15 mm), and ventral premotor cortex (PMv; AP ~20 mm, ML ~20 mm). After dural resection, a custom-made moveable microwire array^39^ was placed over each area. Each array consisted of 12 tungsten microwires (50 μm diameter, impedance 100–200 kΩ at 1 kHz; Advent Research Materials, UK), passing through parallel polyimide guide tubes in two rows of six aligned to the central or arcuate sulcus, with spacing of ~200 μm within rows and ~2 mm between rows. The craniotomy was sealed with cyanoacrylate, gelfoam and dental acrylic, and a titanium casing was attached to protect the microwires and connectors. Animals recovered and returned to training for 1–2 weeks before the microwires were lowered into the cortex. Each microwire was advanced until clear spiking activity was heard. Our use of flexible, moveable microwires, while advantageous for single-unit recording, precluded us from providing accurate data regarding separation of electrode recording sites, since X-ray imaging suggests the electrodes do not necessarily remain parallel as they are advanced. Depending on the experiment, we left the microwires in place up for many months at a time or moved them as often as twice per week to obtain new signals.

### Electrophysiological recordings

The majority of data were acquired using a TDT-RZ2 digital signal processor and acquisition system (Tucker Davis Technologies, FL, USA). Cortical signals were acquired by a digitizing pre-amplifier (48.8 kHz; frequency response 3 dB for 0.35 Hz–7.5 kHz, 6 dB for 0.2 Hz–8.5 kHz) from 24 microwire electrodes, relative to a subdural reference. In experiments involving real-time estimation of firing rates, the *lf–*LFP was extracted by low-pass filtering the raw signal online at 5 Hz (digital biquad filter) and downsampling to 48.8 Hz. Neuronal spiking activity was extracted by digitally band-pass filtering the raw signal (1–8 kHz) and thresholding. We classified single-unit spikes in a semi-supervised fashion using the TDT online PC-based feature extraction and clustering software suite.

Data for Fig. 1a–c and Supplementary Figs 8 and 9 were acquired using a system based around a CED Power-1401 acquisition system (Cambridge Electronic Design, Cambridge, UK), which is described in detail in Supplementary Methods. Both recording systems were used with monkeys A and D. Only the TDT-based system was used with monkey R.

All offline analysis was performed in MATLAB (Mathworks, MA, USA). Channels were excluded from further analysis if visual inspection of the LFP signals indicated that the electrodes or their insulation were damaged (flat signals, consistent wideband noise or large artefacts). If not already extracted online, we extracted *lf*–LFP signals offline by low-pass filtering LFPs at 5 Hz (zero-phase 5th-order Butterworth filter, MATLAB) and downsampling to 48.8 Hz.

### Estimating *lf*–LFPs from neuronal firing rates

The spike events of *P* neurons at time *t* were binned with the same sampling interval as the *lf*–LFP, demeaned and assigned to the *P*-dimensional vector **x**(*t*). *lf*–LFP vectors of *Q* recording channels were demeaned and assigned to the *Q*-dimensional vector **y**(*t*). We used a multiple-input, multiple-output (MIMO) model defined by the equation:




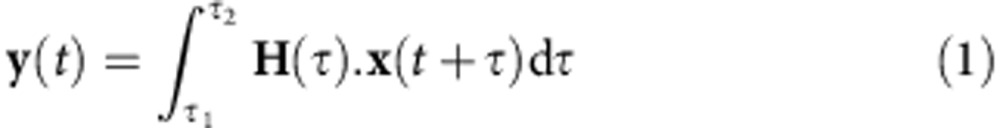




where **H**(*τ*) is an unknown *Q*-by*-P* matrix of finite impulse response (FIR) filter kernels, that are a function of the time interval, *τ*, relative to spike occurrence. For offline *lf*–LFP estimation, we used *τ*_1_=−2.0 s and *τ*_2_=2.0 s. We solved for the filter kernel matrix, **H**(*τ*), of this system using the correlation-based approach of Perreault *et al*.^52^, which is a computationally efficient approximation to least-squares regression under reasonable assumptions. Conceptually, an individual filter kernel element, *h_pq_*(*τ*), from this matrix is very similar to the STA (and looks similar when plotted against *τ*), but it excludes contributions from the auto- and cross-correlation structure within multichannel firing rates.

Next, using these filter kernels, we produced *lf*–LFP estimates, **ŷ**(*t*), from firing rate data according to the same model with:




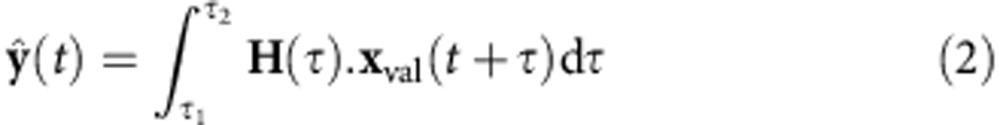




where **x**_val_(*t*) is firing rate data from validation data comprising 25% of the recording. The performance of the model was quantified by the Pearson’s correlation coefficient, *r*, between the estimated *lf*–LFP, *ŷ*_*q*_(*t*), and the actual *lf*–LFP, *y*_*q*_(*t*).

### Estimating neuronal firing rates from *lf–*LFPs

In theory we could apply the MIMO approach described above to solve directly the inverse problem of estimating firing rates from LFPs. However, due to strong correlations between LFP inputs, we found that models fitted to the high-dimensional data suffered from instability and generalized poorly. Therefore we reduced the dimensionality of the LFP data used as input to the MIMO model. A common way to achieve this is by applying PC analysis to the LFP signals. However, the components of the LFP that are most informative about an individual neuron may not be those that capture the greatest overall variance. Instead we used a five-stage approach, guided by the biophysically reasonable assumption that the SRSP associated with each neuron is composed of a discrete number of components:

(1) we built a forward MIMO model (equation 1) with *P* neuronal firing rates, **x**(*t*), as inputs, and *Q lf*–LFPs, **y**(*t*), as outputs, to generate the *Q*-by-*P* matrix of filter kernels, **H**(*τ*). Typically, we estimated two neuronal firing rates simultaneously (Supplementary Fig. 3a–c) and therefore the *lf*–LFPs from both of those electrode channels were excluded from the model. For offline firing rate estimation, we used *τ*_1_=−2.0 s and *τ*_2_=2.0 s.

(2) We performed PC analysis on the filter kernels (Supplementary Fig. 3d). This was motivated by the observation that the variability of the SRSP across LFP channels could be captured by a small number of components (Fig. 3), implying that only a discrete number of sources within the LFP are informative of the spiking of a given neuron. The first six SRSP-PCs were used for the remainder of the analysis, yielding **h′**_*p*_(*τ*), a vector of six filter kernels where the subscript indicates that these are appropriate for estimating cell *p.* (Note that in Fig. 5a–d only three PCs were used, while in Fig. 5e,f the number of PCs used was a dependent variable.)

(3) At this stage it would be possible to project the LFP directly onto the six SRSP-PC axes to achieve dimensionality reduction. However, such an approach would be suboptimal since, while these projections maximize the information about a given neuron, they do not minimize uncorrelated noise (which would not appear in the SRSP). Instead, for each neuron we first obtained a ‘source estimate’ vector, **s**_*p*_(*t*), (Supplementary Fig. 3e) by convolving the firing rate *x_p_*(*t*) with the SRSP-PC kernels:




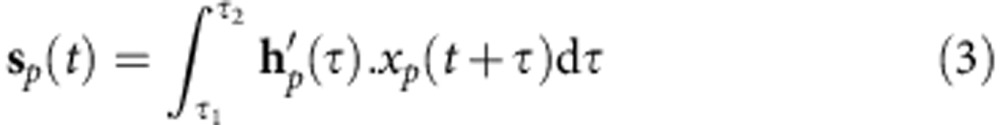




(4) We then found the projection of the LFP data that best approximated this source estimate. Linear regression was performed between the source estimates, **s**_*p*_(*t*), and the *Q lf–*LFPs, **y**(*t*), yielding a *Q*-by-6 ‘weighting matrix’ **M**_*p*_ (Supplementary Fig. 3h) to transform the *lf*–LFP data into a six-dimensional ‘source projection’ vector, **y′**_*p*_(*t*), that best fitted the six source estimates of neuron *p* (Supplementary Fig. 3f).

(5) For each neuron, we calculated an ‘inverse filter’ kernel vector, **κ**_*p*_(*τ*), to deconvolve the source projections for neuron *p* and produce an estimated firing rate (Supplementary Fig. 3g). To do this we fitted a new model, with source projections, **y′**_*p*_(*t*), as inputs and the low-pass-filtered (5 Hz; zero-phase 5th-order Butterworth) actual firing rate, *x*_*p*_(*t*), as an output:




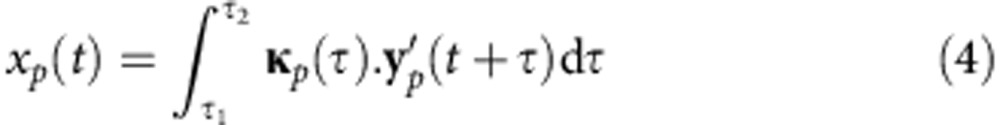




We found that the stability of this model (an inverse Wiener–Kolmogorov filter) could be improved by adding low-amplitude Gaussian noise (mean=0; 
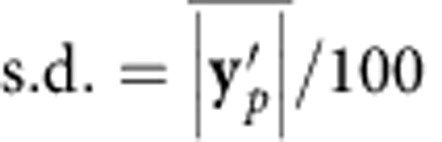
) to each source projection before fitting, which is equivalent to adding a regularization term to penalize the sum-square of the filter kernels.

An ‘*lf–*LFP decoder’ for neuron *p* thus consisted of two elements: (1) the *lf*–LFP weighting matrix, **M**_*p*_; and (2) the inverse filter kernels, **κ**_*p*_(*τ*). Both were stored for later use in either online or offline firing rate estimation. To test the model, validation *lf*–LFP data, **y**_val_, (either 25% of the recording, or data from another behavioural task/another day) were transformed by the weighting matrix, **M**_*p*_, and deconvolved using the inverse kernels to produce a firing rate estimation (Supplementary Fig. 3i) for a particular neuron:




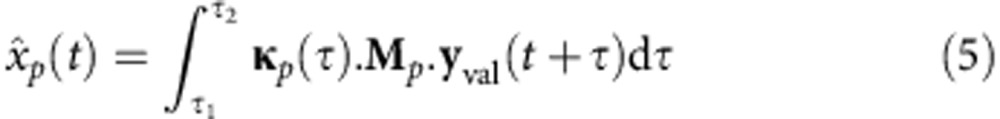




### Online firing rate estimation

For online firing rate estimation during the ‘LFP-control’ BMI task, we used the same approach as offline, but with *τ*_1_=−1.8 s and *τ*_2_=0.2 s, such that online firing rate estimation was delayed by 0.2 s relative to the real-time data. Projection of *lf*–LFP signals into the source space and subsequent inverse filtering were implemented by hardware digital signal processors within the TDT-RZ2 using the TDT Real-time Processor Visual Design Studio (RPvdsEx) software.

### Assessing model performance

The quality of firing rate estimation was quantified for each neuron using the Pearson correlation coefficient, *r*, between the low-pass-filtered actual firing rate, *x*_*p*_(*t*), and the estimated firing rate, 
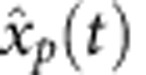
 over validation data. Since successive samples of *lf–*LFPs or firing rates are not independent, the statistical significance of *r*-values (between estimated and actual data) cannot be inferred from parametric assumptions. Therefore we estimated the distribution of *r*-values under the null hypothesis of no relationship by shifting (with circular wrapping) the actual and estimated data by all possible time-lags >5 s. The reported *r*-value was considered significant (*P*<0.05, two-tailed) if it fell above the 97.5th centile of the resulting distribution. In the figures, firing rate estimations whose *r*-values fell above this threshold are shown as filled circles and those below as empty circles. In Fig. 6a–d, the mean (±s.e.m.) of the significance threshold across all neurons is shown for illustrative purposes only, although each neuron estimate underwent individual significance testing.

We also calculated correlation in the frequency domain using Welch’s coherence estimate (‘mscohere.m’ in MATLAB Signal Processing Toolbox), with a 128-point fast Fourier transform with non-overlapping windows. Significance was determined according to ref 53:




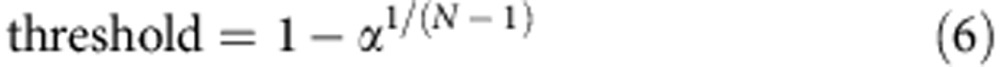




where the *N* is the number of disjoint windows (193 in the example shown in Supplementary Fig. 4b) and the significance level *α*=0.05.

### Performance with increasing numbers of neurons/*lf–*LFPs/PCs

To determine how the quality of *lf*–LFP fit depended on the size of the neuronal sample (Fig. 2), every *lf*–LFP channel was estimated using 120 random draws of *P* neurons increasing from one up to the number of neurons available. To determine how the quality of firing rate fit depended on size of the *lf*–LFP sample (Fig. 5a–d), every neuron’s firing rate was estimated using 120 random draws of *Q lf*–LFPs increasing from three up to the number of channels available (excluding that recorded on the same electrode as the neuron of interest) and applying dimensionality reduction based on three SRSP-PCs. For comparison, we also estimated each neuron’s firing using all available *lf*–LFPs, not excluding that on the same channel as the neuron (these results are shown as open squares in Fig. 5a–d). To determine how the quality of firing rate fit depended on the number of SRSP-PCs (Fig. 5e,f), every neuron’s firing rate was estimated using all *lf*–LFPs (excluding that recorded on the same electrode as the neuron of interest) projected into a source estimate space with dimensionality increasing from one up to the number of channels available (that is, no dimensionality reduction). The quality of fit was compared against models based on increasing numbers of *lf*–LFP PCs from one up to the number of channels available. In each of the figures, error bars show s.e.m. across different channels, and are therefore are not artificially reduced by the large number of permutations averaged to estimate the mean for each channel.

### Assessing estimation of neural population components

For the simultaneous estimation of all firing rates, we included *lf*–LFPs on the same channels as the estimated neurons, since there would otherwise have been insufficient *lf*–LFPs available. PC analysis was performed across the low-pass-filtered firing rates, **x**(*t*), of all neurons recorded in the session (the *P*-dimensional ‘neural space’), producing neural PCs, 
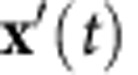
. Estimated firing rates of the same neurons, 
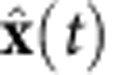
, were reprojected onto the same PC axes, producing component estimates, 
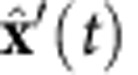
. Comparison between 
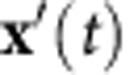
 and 
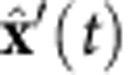
 used the correlation coefficient, *r*, with bootstrap significance testing (as above).

### Tuning index for the ‘LFP-control’ BMI task

The actual firing rate of each cell was normalized to zero mean and unity variance over the ‘LFP-control’ BMI session. Firing rate profiles for each target were aligned to the end of successful trials and averaged separately for estimated and all other neurons. The task-modulation of firing rates was quantified using a tuning index, calculated for each neuron during the hold period of the task (adjusted for the 0.2 s delay in the firing rate estimation), according to:




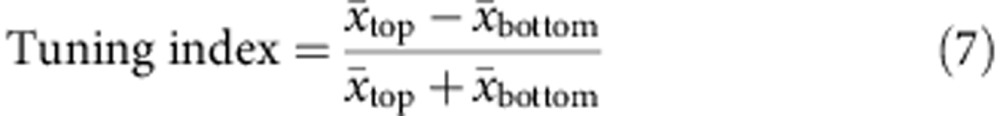




where 
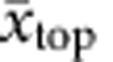
 and 
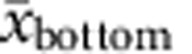
 are the mean firing rates of the neuron during the (adjusted) hold period across all trials for the top target and bottom target, respectively. The tuning index distributions of estimated (controlling) neurons versus all other (non-controlling) neurons were compared using the Mann–Whitney *U*-test implemented in SPSS (IBM, NY), because data did not pass normality tests (Shapiro–Wilk, *P*<0.05).

## Additional information

**How to cite this article:** Hall, T. M. *et al*. Real-time estimation and biofeedback of single-neuron firing rates using local field potentials. *Nat. Commun.* 5:5462 doi: 10.1038/ncomms6462 (2014).

## Supplementary Material

Supplementary Information Supplementary Figures 1-9, Supplementary Methods and Supplementary Discussion
