# Supplementary material for: Real-time estimation and biofeedback of single-neuron firing rates using local field potentials
Source: Nat Commun. 2014 Nov 14;5:5462. doi: 10.1038/ncomms6462 (PMC4243238; doi:10.1038/ncomms6462)
Supplement: Supplementary Information — Supplementary Figures 1-9, Supplementary Methods and Supplementary Discussion [file ncomms6462-s1.pdf]

Real-time estimation and biofeedback of single-neuron firing rates using local field potentials

Thomas M Hall, Kianoush Nazarpour and Andrew Jackson

Supplementary Figures

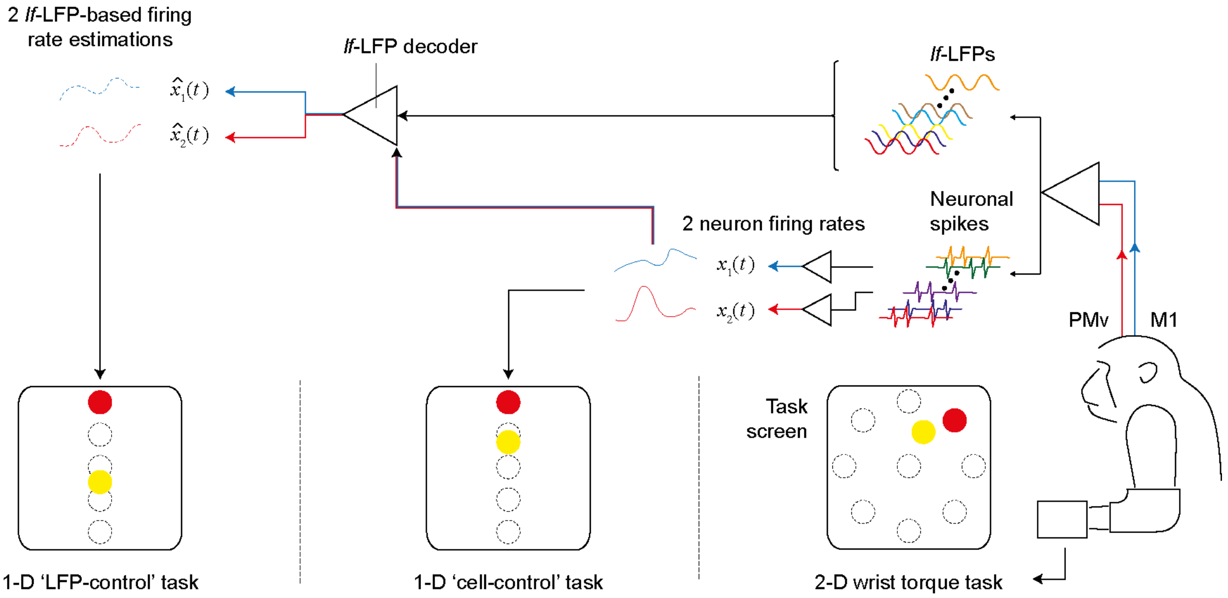

Supplementary Figure 1 | Experimental setup. Schematic illustration of the three tasks performed by the monkeys: the torque task, 'cell-control' BMI task and 'LFP-control' BMI task. See **Methods** and **Supplementary Methods** for full description.

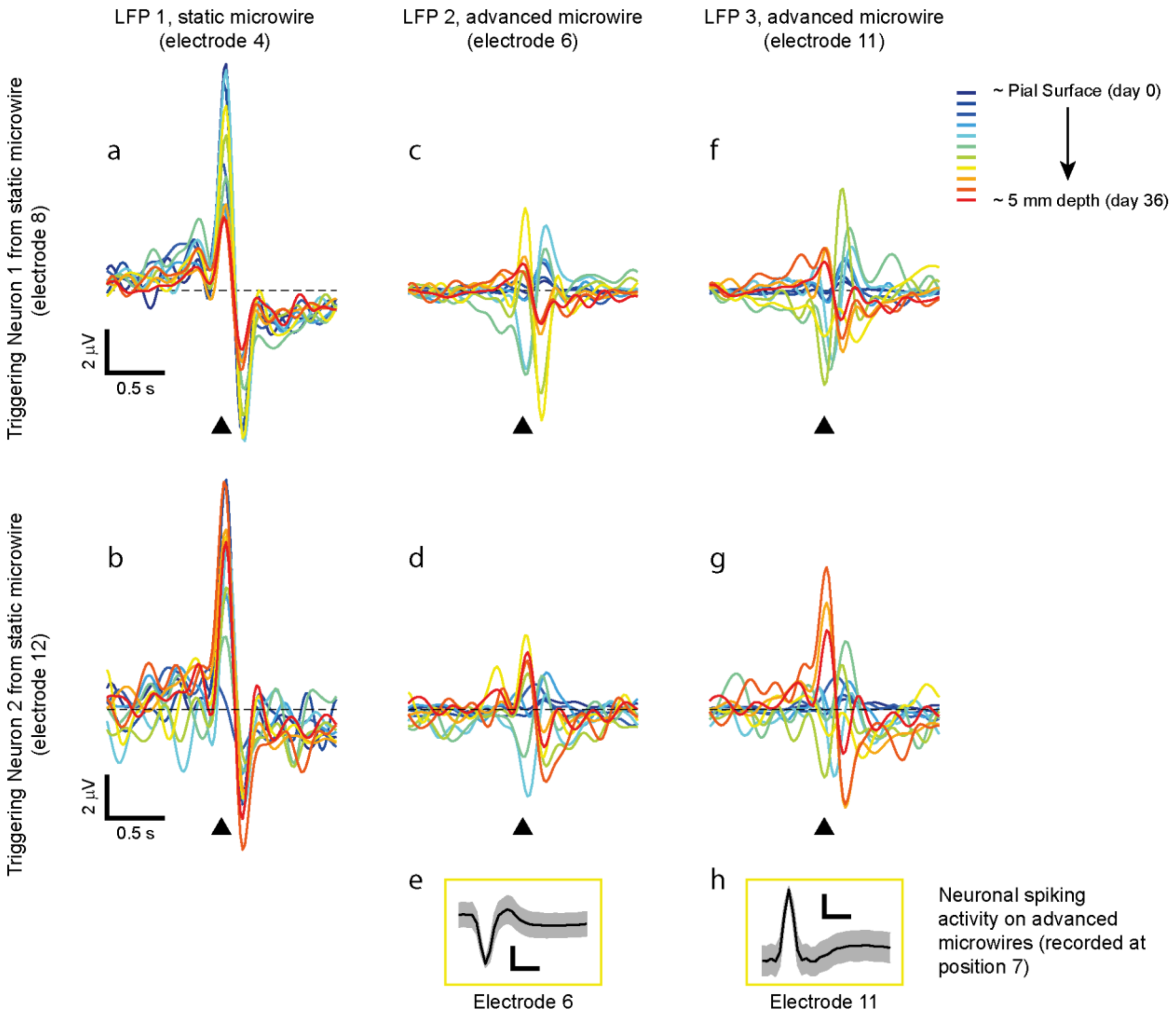

Supplementary Figure 2 | Polarity inversion of SRSPs with increasing cortical depth. Two single neurons were recorded over thirty-six days from static microwires in M1 of Monkey D. *Lf*-LFP recordings were concurrently recorded from a static microwire located nearby in M1, and from two further M1 microwires whose depths were advanced manually by approximately 0.5 mm every 3 to 4 days under sedation. The polarity and shape of the SRSP from the static *lf*-LFP electrode (**a-b**) remained highly consistent over time (with some minor variation in SRSP amplitude). In contrast, the SRSP on the advanced electrodes (**c-d, f-g**) gradually increased in amplitude with depth, before abruptly inverting polarity, then subsequently reducing in amplitude. Black arrows (▲) indicate time of triggering spike. Simultaneous spike recording from the advanced electrodes confirmed that the polarity inversion occurred within the cortical grey matter. For example, spike shapes are shown (**e,h**) from both advanced electrodes at position 7 (~ 3.5 mm depth, position corresponding to yellow line in the above panels); scale bars indicate 0.25 ms and 10 µV.

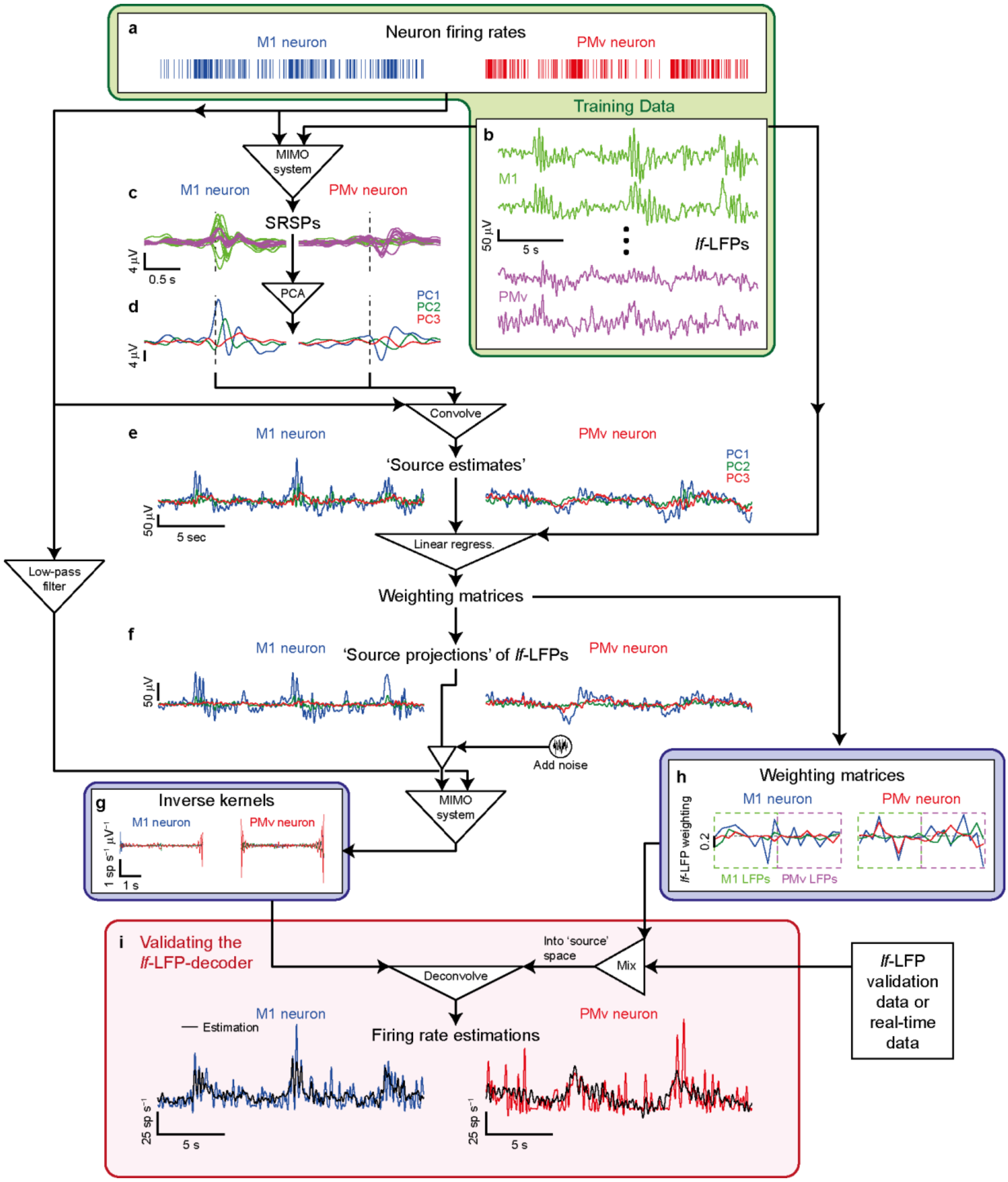

Supplementary Figure 3 | Method for estimating firing rates from *lf*-LFPs. Schematic illustration of the steps used to estimate the firing rates of two neurons (see **Online Methods** for description of steps and symbols used). *Green box* represents training data. *Blue boxes* are the two elements of the linear model. *Red box* represents validating the model. (**a**) Example rasters of an M1 neuron and PMv neuron. Binned firing rates (x_1 and x_2) were calculated and used for the succeeding steps. (**b**) Four of the 20 *lf*-LFPs (**y**) used in the model (22 in total, minus those recorded on the same channels as the estimated neurons). (**c**) SRSP kernels ($\mathbf{h}_1$ and $\mathbf{h}_2$) for each neuron. (**d**) SRSP-PC kernels ($\mathbf{h'}_1$ and $\mathbf{h'}_2$;

first three shown). (**e**) 'Source estimates' ($\mathbf{s}_1$ and $\mathbf{s}_2$) for each SRSP-PC. (**f**) 'Source projections' ($\mathbf{y'}_1$ and $\mathbf{y'}_2$) of the *lf*-LFP that best fit the source estimates. (**g**) Inverse filter kernels ($\boldsymbol{\kappa}_1$ and $\boldsymbol{\kappa}_2$) for each neuron. (**h**) Weighting matrices ($\mathbf{M}_1$ and $\mathbf{M}_2$) for generating the *lf*-LFP source projections for each neuron. (**i**) Firing rate estimates ($\hat{x}_1$ and $\hat{x}_2$) for each neuron based on deconvolution of source projections, compared against low-pass filtered actual firing rates.

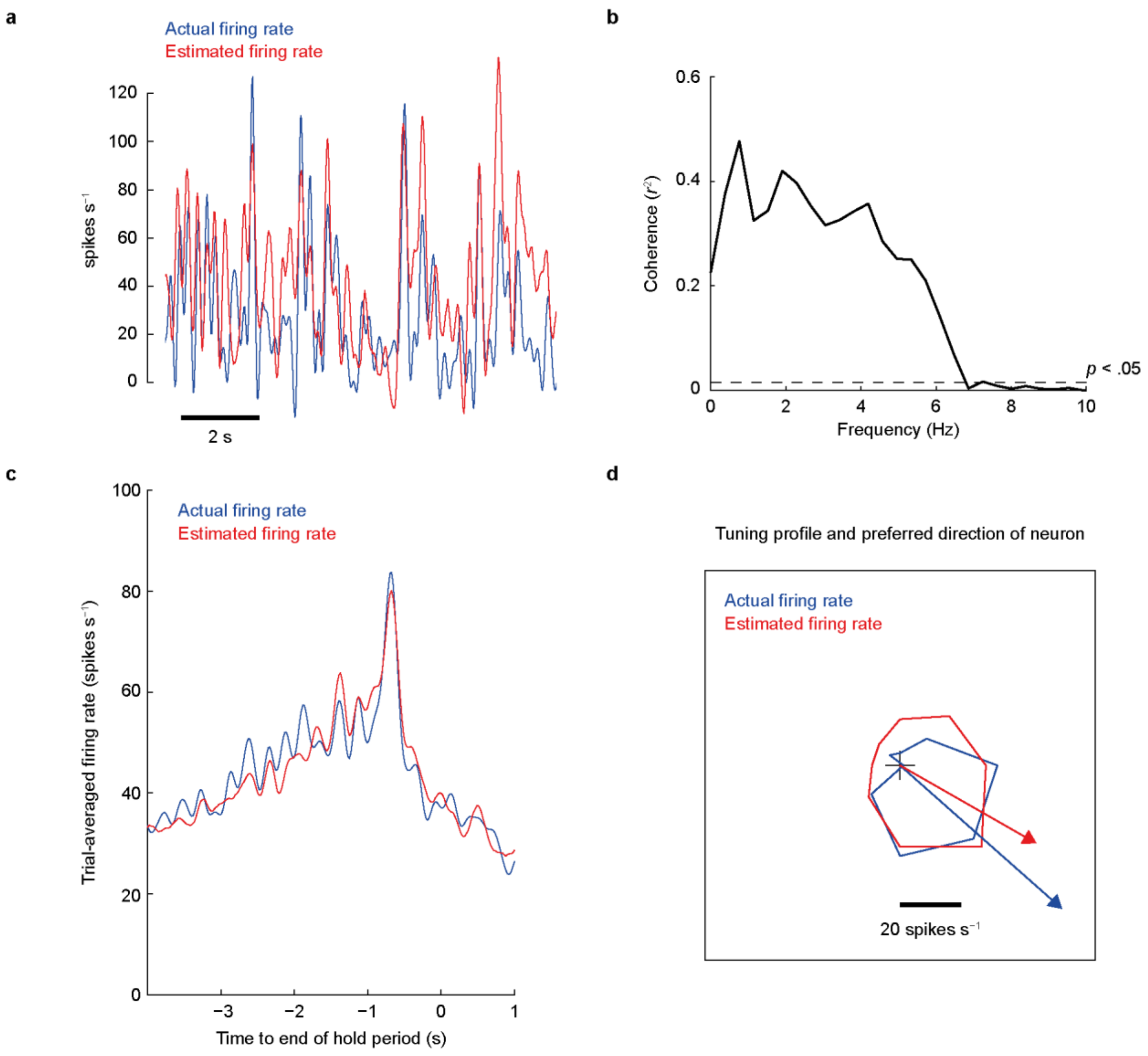

Supplementary Figure 4 | Coherence and task-relationship of actual and estimated firing rates. (**a**) Typical example, showing actual (*blue*) and estimated (*red*) firing rates for an M1 neuron (from Monkey D) during the torque task, estimated using a model based on 22 *lf*-LFPs. (**b**) Coherence between actual and estimated firing rate with a significance threshold (dashed line) at $p < .05$. (**c**) Mean firing rate across trials of an example M1 neuron (from Monkey D) aligned to the end of the 'hold' period of the torque task (55 trials). *Blue* line shows the trial-averaged actual firing rate (low-pass filtered at 5 Hz). *Red* line shows the trial-averaged LFP-based estimate for the same neuron. (**d**) Polar plot of actual and estimated firing rates for different target positions (average of 1 s prior to the end of the hold period, with baseline firing rate subtracted). Also shown is the 'preferred direction', calculated as the vector sum of the tuning profile.

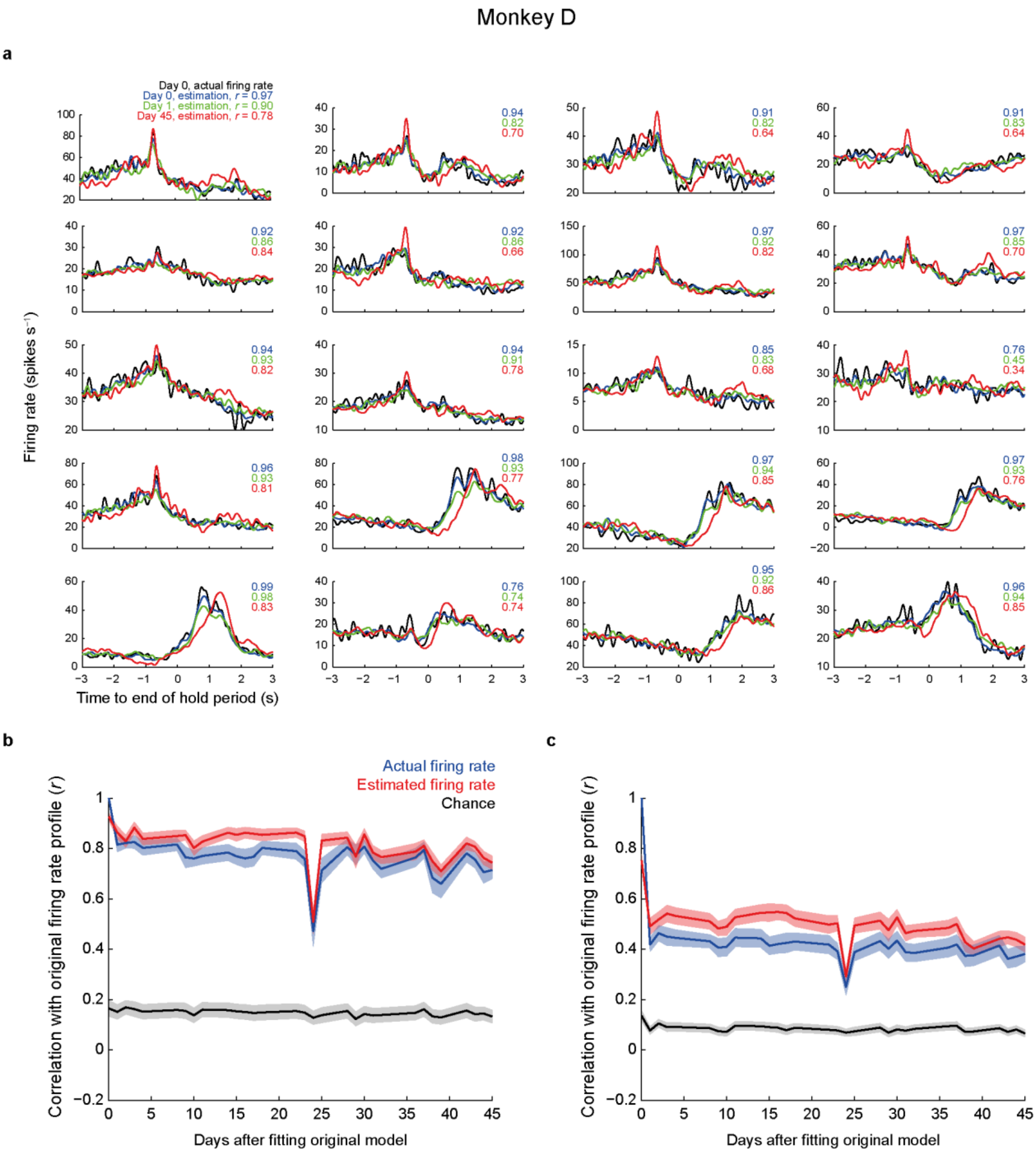

Supplementary Figure 5 | Actual and estimated trial-aligned firing rate profiles in Monkey D. (**a**) Each sub-panel shows data for one of the 20 neurons from the sessions shown in **Fig. 6a**. In each subpanel: the *black line* shows the mean trial-aligned actual firing rate profile averaged over ~ 50 trials of the torque task (including all eight target directions) on day zero; the *blue line* shows trial-averaged firing rate estimates derived from *lf*-LFP for the same trials; the *green line* shows the trial-averaged *estimated* firing rate profile for trials performed one day later; the *red line* shows the trial-averaged *estimated* firing rate profile on day 45. Inset numbers indicate the correlation coefficient

(*r*) between the actual firing rate profile on day 0 and the estimated firing rate profile. Firing rates were estimated using model parameters fit to the data on day 0. PMv neurons were commonly most modulated at the end of the trial, when the subject took food reward with the ipsilateral (right) hand. (**b**) *Red line* shows the mean (n = 20 neurons; shading indicates ± s.e.m.) of the correlation coefficient (*r*) between the trial-aligned *actual* firing rate profile on day zero and the trial-aligned LFP-based *estimate* of the firing rate profile on successive days. *Blue line* shows the mean (± s.e.m.) correlation coefficient of the actual trial-aligned firing rate profile on day zero and the *actual* trial-aligned firing rate profile on successive days. *Black line* shows the mean (± s.e.m.) correlation between the trial-aligned actual firing rate profile on day zero and the trial-aligned LFP-based estimate of the firing rate profile of a *different neuron* on successive days (i.e. the correlation that would be expected by chance if the LFP-based estimate did not capture firing rate modulations specific to the estimated neuron). Note that variation in the monkey's behaviour from one day to the next limits the accuracy with which trial-averaged profiles on subsequent days resemble day zero, but this affects both real and estimated firing rate data equally (e.g. the downwards spike on day 24). In general, the LFP-based estimate performs at least as well as the actual firing rate throughout the recording, and is significantly greater than chance performance. (**c**) Shows the same as panel **b**, but for trials separated according to target direction (~ 6 trials each for the 8 circumferential targets in the torque task). Correlation coefficients are calculated across the concatenation of firing rate profiles for the individual targets.

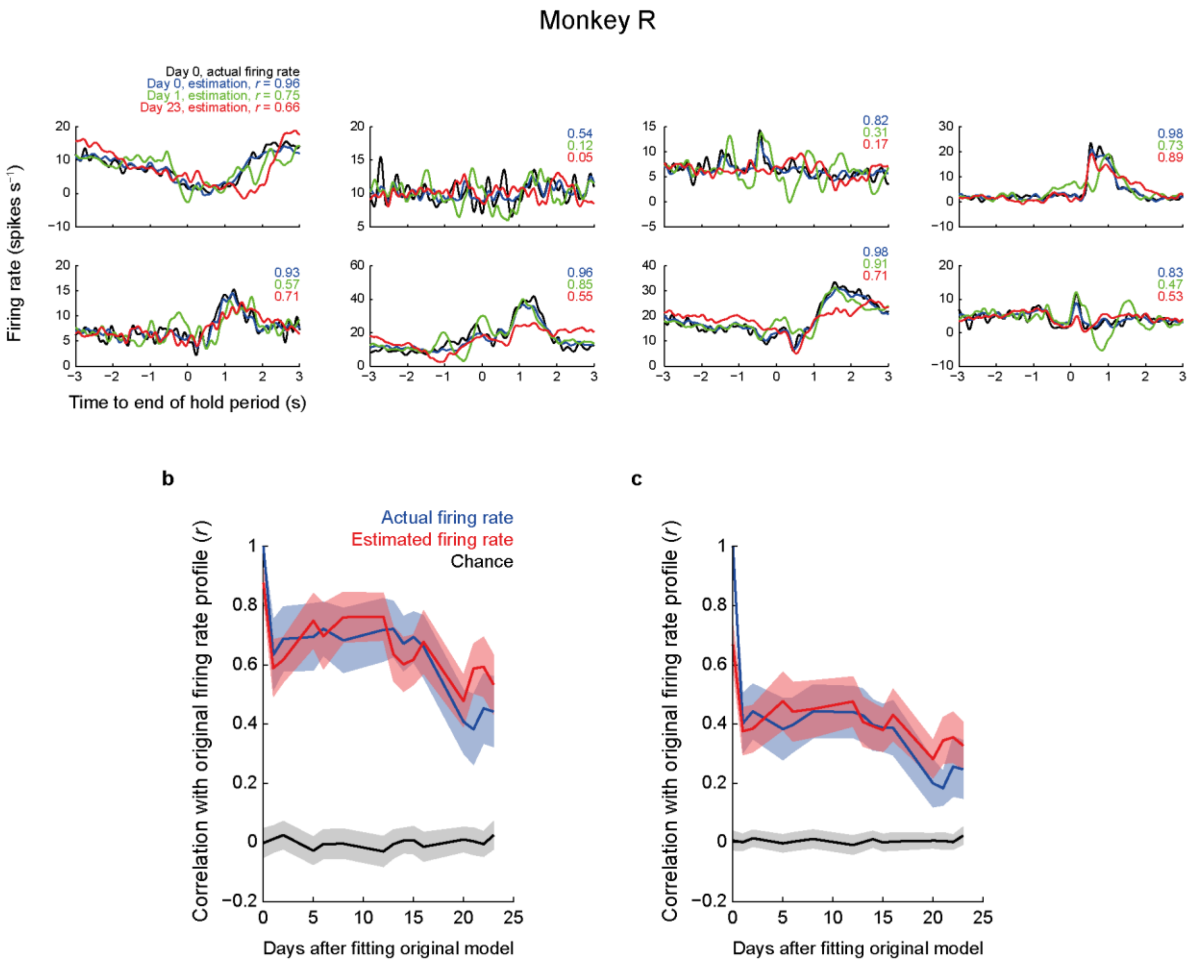

Supplementary Figure 6 | Actual and estimated trial-aligned firing rate profiles in Monkey R. This figure shows the same as **Supplementary Fig. 5**, but for the neurons shown in **Fig. 6b**, recorded in Monkey R over 23 days (n = 8 neurons; shading indicates ±s.e.m.).

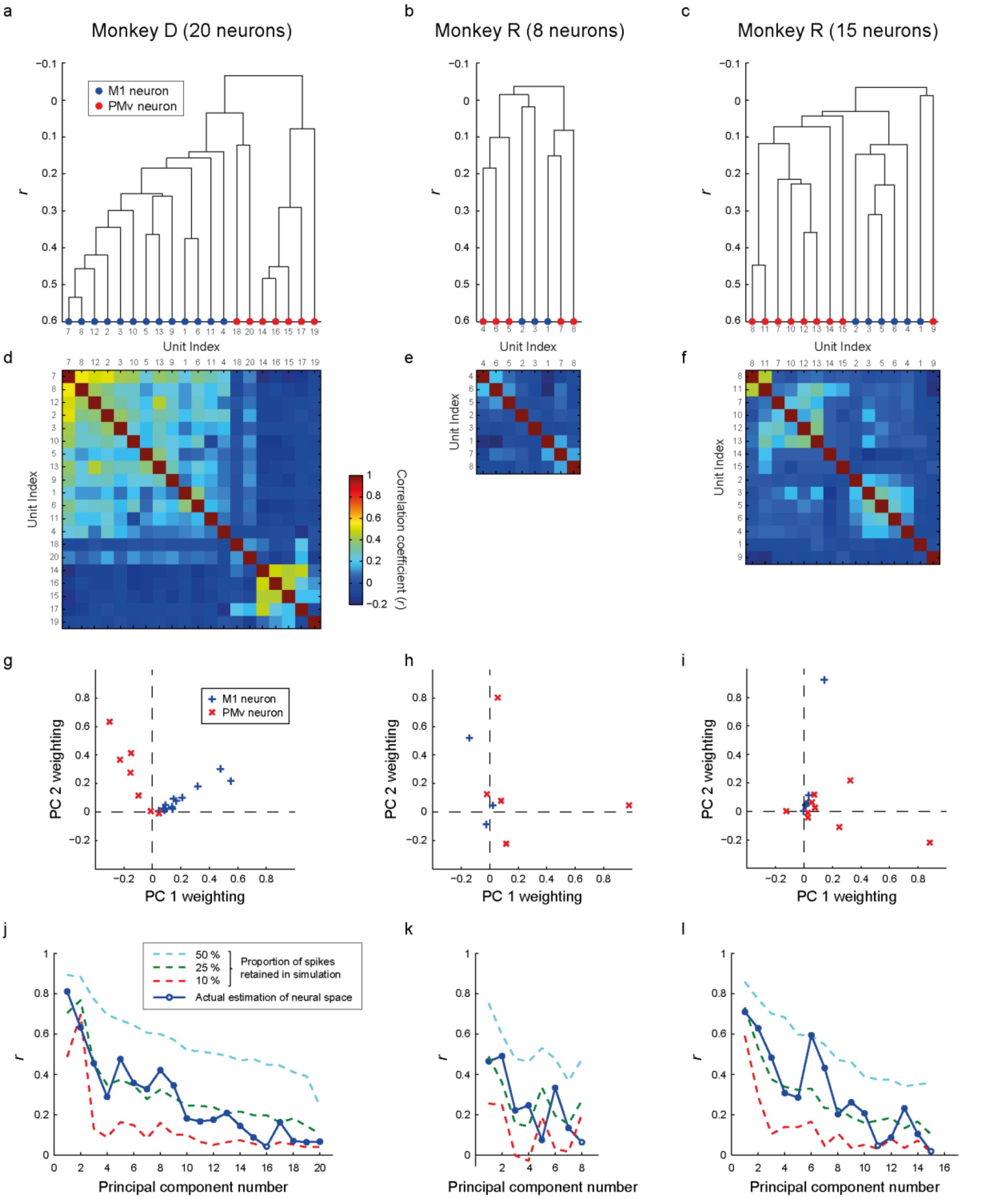

Supplementary Figure 7 | Clustering and PC analysis of the neural firing rate-space. (**a**) Dendrogram showing a hierarchical cluster tree based on correlations between the firing rates of 20 neurons in Monkey D (Same data as in **Fig. 2a,c**, **Fig. 5a,c,e** and **Fig. 6e**). (**b**) Same for 8 neurons in Monkey R (same data as in **Fig. 6f**). (**c**) Same for another dataset in Monkey R with 15 neurons (same data as in **Fig. 2b,d** and **Fig. 5b,d,f**). Note that in all datasets, neurons within the same cortical area tend be located on neighbouring branches of the tree. (**d-f**) Full correlation matrix for the same

datasets, ordered according to the corresponding cluster tree. Broad correlations can be seen across many neurons within the same cortical area. However, there is not clear evidence for highly-correlated subsets of neurons within these broad groups. (**g-i**) Scatter plot of weightings for PC1 and PC2 of the high-dimensional neural space for the same datasets. (**j-l**) Simulation showing the effect of artificially introducing noise into the actual spike recordings by mixing a proportion of spikes between spike trains (see **Supplementary Methods**). *Dashed lines* show the effect of retaining 50%, 25% or 10% of spikes in the simulation. The drop-off seen in our ability to estimate higher order PCs of the neural space from *lf*-LFPs (*solid blue* line, see also **Fig. 6e,f**) is comparable to estimates based on retaining 25% of the actual spike trains for each neuron. Filled circles indicate significant estimates ($p < .05$, two-tailed; non-parametric bootstrap).

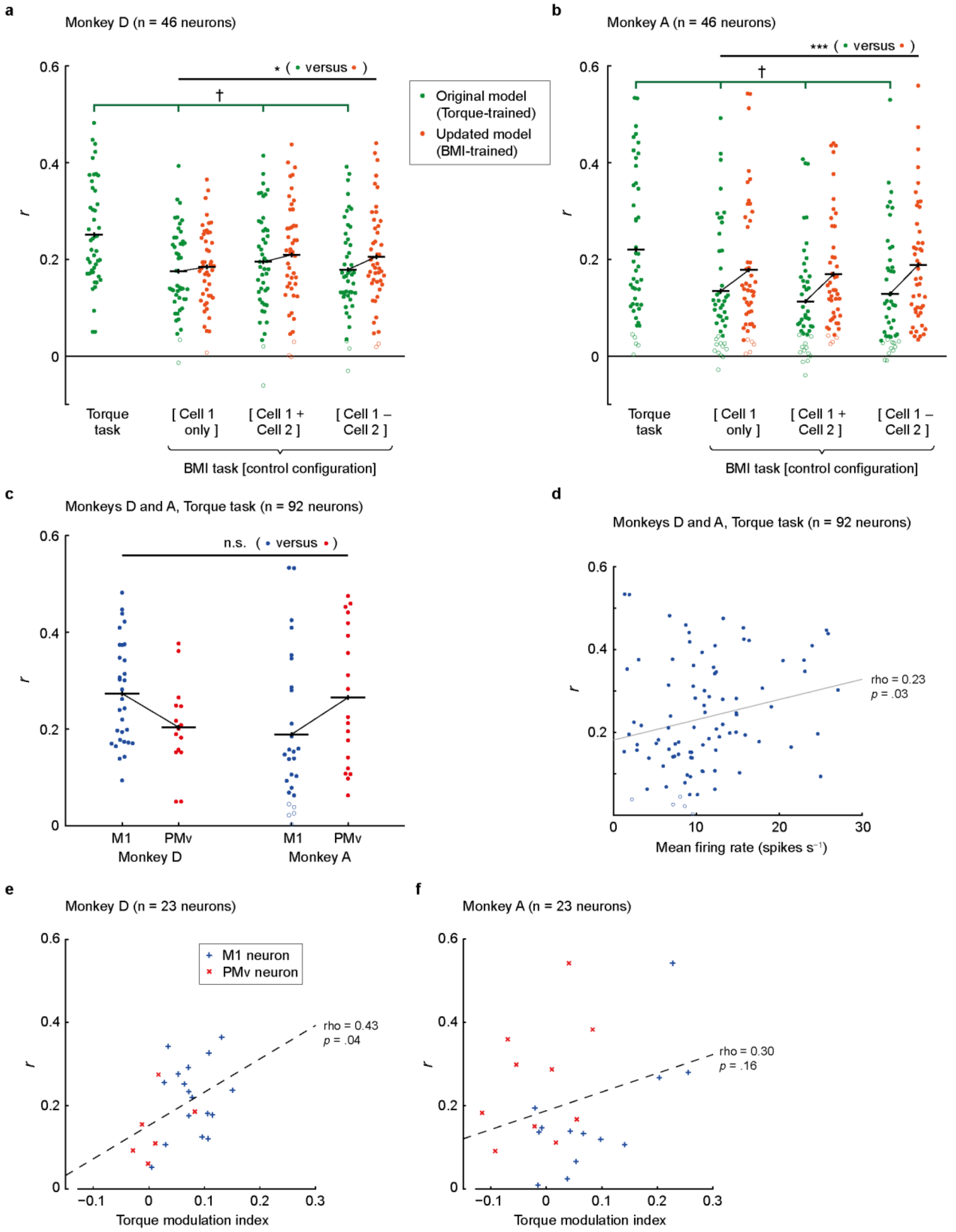

Supplementary Figure 8 | Generalisation of *lf*-LFP-based firing rate estimate across 'cell-control' BMI tasks. (**a**) Performance (*r*) of firing rate estimation using a model built on the torque task data (*green*, 'Torque-trained') tested on validation data from three different 'cell-control' BMI tasks. Also shown is performance of model built on data from the same BMI condition as the validation data

(*orange*, 'BMI-trained'). Data from Monkey D; 23 experiments with 2 neurons per experiment; 31 M1 and 15 PMv neurons in total. (**b**) The same for Monkey A, 23 experiments with 2 neurons per experiment; 27 M1 and 19 PMv neurons. (**c**) Comparison of model performance (*r*) for M1 and PMv neurons during the torque task. In panels **a-c**: black horizontal lines indicate group means; *, $p < .05$ by two-way repeated-measures (*rm-*) ANOVA; ***, $p < .001$ by two-way *rm*-ANOVA; †, $p < .001$ by one-way *rm*-ANOVA; *n.s.*, not significant by two-way independent samples ANOVA ($p > .05$ and $F < 1$). See **Supplementary Discussion** for further description of statistics. (**d**) Estimation performance (*r*) vs. mean firing rate for all 92 neurons (46 neurons from each subject). Dashed shows simple least-squares regression line, with correlation coefficient, *rho* and *p*-value. In panels **a-d**, open circles indicate individual neurons whose firing rate estimation was non-significant by non-parametric bootstrap test ($p < .05$, two-tailed) (**e**) Performance (*r*) of firing rate estimation vs. torque modulation index (TMI) for the 'Cell 1 only' cell-control BMI task in Monkey D ($n = 23$ neurons). (**f**) Same for Monkey A ($n = 23$ neurons). Data are plotted separately for M1 (*blue* '+') or PMv (*red* '×') neurons. Dashed line shows least-squares regression through all points with correlation coefficient, *rho*, and *p*-value.

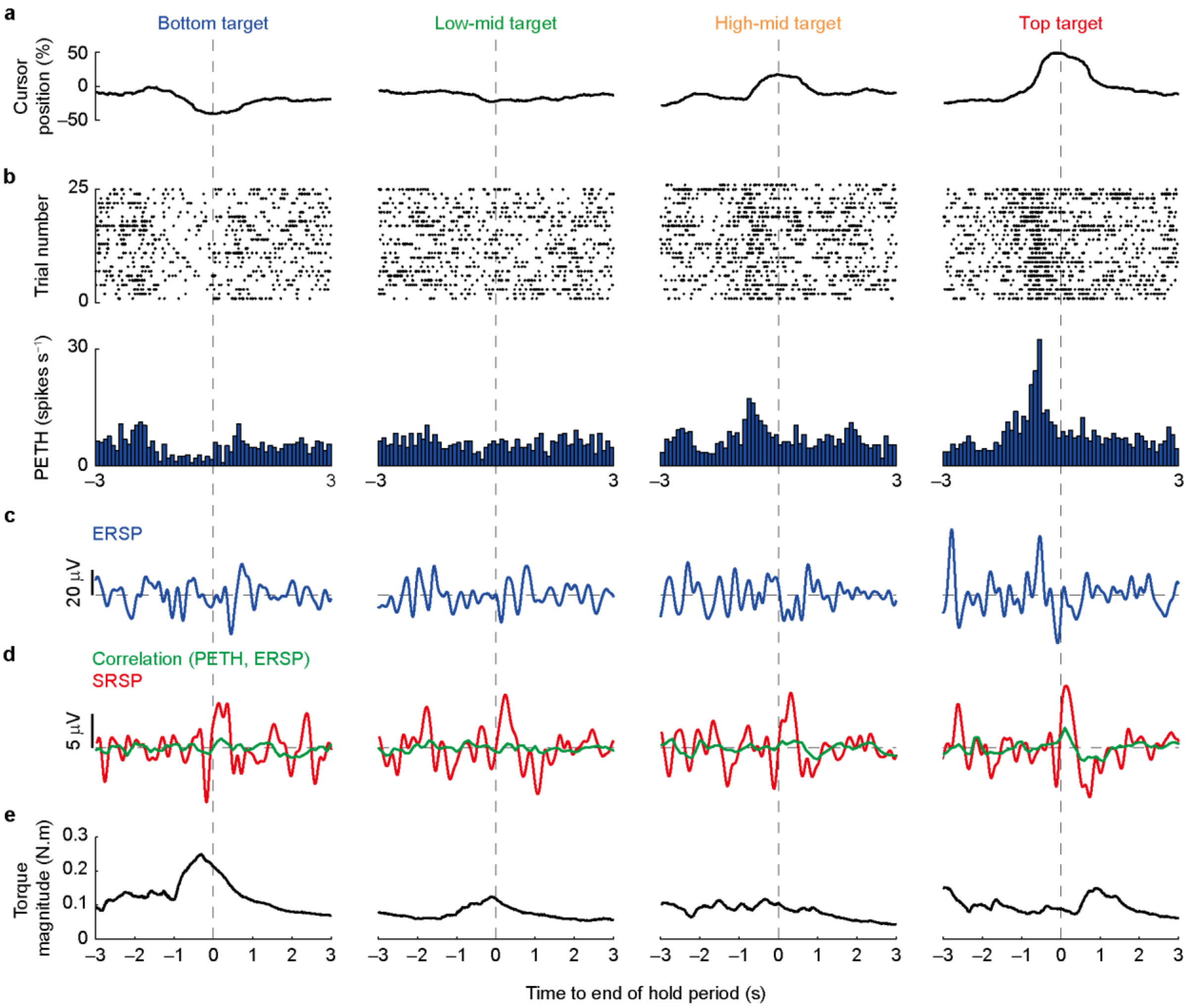

Supplementary Figure 9 | SRSP during 'cell-control' BMI task. (**a**) Trial-averaged cursor position, aligned to end of hold period, during the 1-D 'cell-control' BMI task in which Monkey A controlled the firing rate of a single PMv neuron (see **Supplementary Methods**). (**b**) Spike raster and peri-event spike time histogram (PETH) of the controlling neuron. (**c**) Trial-aligned average of an *lf*-LFP on another PMv channel, labelled as the 'event-related slow potential' (ERSP). (**d**) *Red*, spike-related slow-potential (SRSP) of the *lf*-LFP for only those spikes shown above. *Green*, cross-correlation of the ERSP and PETH shown above. (**e**) Trial-aligned average wrist torque magnitude. See **Supplementary Discussion** for further details.

Supplementary Methods

'Cell-control' BMI task. In this task, 1-D cursor position was controlled by actual firing rates of neurons. Two neurons were discriminated during the 'torque task'. The smoothed instantaneous firing rate (x) of each was calculated in real-time by convolution of spike times with a rectangular window (width 400 ms). Each firing rate was mapped to normalized screen co-ordinates using the median and $5^{th}/95^{th}$ centiles of its distribution (as described for the 'LFP-control' task). Within a day's experiment, for blocks of 100 trials each, one-dimensional cursor position, c, was controlled by the firing rate of one cell, $x_{1(\mathrm{norm})}$ or by the summed ($[x_{1(\mathrm{norm})} + x_{2(\mathrm{norm})}]/\sqrt{2}$) or differential ($[x_{1(\mathrm{norm})} - x_{2(\mathrm{norm})}]/\sqrt{2}$) firing rates of the two neurons, where the factor of $1/\sqrt{2}$ was used to make all targets equidistant from the origin in the 2-D normalised neural space. Data from the 'cell-control' BMI task are presented in **Supplementary Figs. 8-9**.

Electrophysiological recordings using the CED-based system. Data for **Fig. 1a-c** and **Supplementary Figs. 8-9** were acquired using a system based around a CED Power-1401 acquisition system (Cambridge Electronic Design, Cambridge, UK). Signals were amplified and filtered into LFP and spike bands using two MPA8I headstages and a PGA1632 amplifier (Multichannel Systems, Reutlingen, Germany). LFP signals were amplified (gain 5000) and band-pass filtered (1 Hz to 300 Hz) before being sampled (1000 Hz) by the Power-1041. Spike activity was amplified (gain 10000), band-pass filtered (300 Hz to 8 kHz) and sampled (18.5 kHz), after which single-unit spikes were classified in a supervised fashion using the template-based online spike sorter of the Power-1401.

Simulating effect of noise on neural component estimation. To simulate the effect of lower signal-to-noise on the ability to estimate higher PCs in the neural space, we introduced

artificial noise as follows. Using a recording with a total of P neurons, we retained a proportion, α, (either 50%, 25% or 10%) of spikes selected randomly for each neuron. We then inserted a proportion, $(1-\alpha)/(P-1)$, of spikes from every other neuron into the spike train. This process was repeated for all P neurons, after which we calculated the low-pass-filtered (5 Hz as previously) and demeaned firing rates for each of the actual and 'noisy' spike trains. We then calculated the correlation coefficient (r) between actual and 'noisy' firing rates along each of the PC axes of the neural space.

Clustering of the neural space. To examine whether subsets of highly-correlated cells were present in the data, hierarchical cluster analysis was performed on the neural space, $\mathbf{x}(t)$, using one minus the sample correlation ($1-r$) with the unweighted average distance (UPGMA) algorithm to measure the distance between clusters (using 'linkage.m' in MATLAB Statistics Toolbox). The resulting dendrogram was used to determine the order of cells in plots of the full pair-wise cross-correlation matrix between firing rates. In such plots, tightly-correlated ensembles would appear as clusters of high correlation values close to the main diagonal.

Torque modulation index for 'cell-control' BMI task. The torque modulation index (TMI) quantified the degree of modulation of wrist torque across each of the four targets during the cell-control BMI task. It was calculated from the gradient of linear regression of average absolute torque (during the hold period) against target position (in screen coordinates). TMI was normalised by the torque-position gain during the torque control task. Thus a TMI value of less one indicates that during cell control, the monkey modulated torque less to acquire peripheral targets than during the torque task, while a value of zero indicates no overall modulation of torque for different targets.

Supplementary Discussion

Generalisation of *lf*-LFP-based firing rate estimation across 'cell-control' BMI tasks. (**Supplementary Fig. 8**). We were interested to see whether the relationship between *lf*-LFPs and neural firing was preserved across tasks that required the generation of specific patterns of neural activity (rather than torque) to successfully acquire targets. Therefore, we built a model based on data collected during the torque task using up to 13 *lf*-LFPs to estimate the firing rates of two neurons (recorded on different electrodes). Significant estimation (at $p < .05$ using the bootstrap test described in **Online Methods**) was achieved for 46/46 neurons in monkey D (mean $r = 0.251$, s.e.m. = 0.016) and 41/46 neurons in monkey A (mean $r = 0.221$, s.e.m. = 0.023). We then assessed the performance of this model on data recorded during 'cell-control' tasks (**Supplementary Fig. 8a,b**). In monkey D, significant estimation was achieved for 43/46 (Cell 1 – Cell 2) and 44/46 (Cell 1 only; Cell 1 + Cell 2) neurons. In monkey A, significant estimation of 34/46 (Cell 1 only; Cell 1 + Cell 2) and 35/46 (Cell 1 – Cell 2) neurons was achieved. In both animals there was a small but significant drop in the quality of estimation, r (Monkey D: Cell 1 only: mean $r = 0.175$, s.e.m. = 0.013; Cell 1 + Cell 2: mean $r = 0.195$, s.e.m. = 0.016; Cell 1 – Cell 2: mean $r = 0.180$, s.e.m. = 0.014; one-way repeated-measures ANOVA (with Greenhouse-Geisser correction), $F_{2.7,120.7} = 23.6$, $p < .001$. Monkey A: Cell 1 only: mean $r = 0.135$, s.e.m. = 0.018; Cell 1 + Cell 2: mean $r = 0.113$, s.e.m. = 0.016; Cell 1 – Cell 2: mean $r = 0.129$, s.e.m. = 0.018; one-way repeated-measures ANOVA, $F_{3,135} = 24.3$, $p < .001$). When compared with models built using data from the same BMI task, the decrease in performance was also small but significant (Monkey D: Cell 1 only: mean $r = 0.184$, s.e.m. = 0.012; Cell 1 + Cell 2: mean $r = 0.209$, s.e.m. = 0.016; Cell 1 – Cell 2: mean $r = 0.206$, s.e.m. = 0.015; two-way repeated-measures ANOVA: effect of torque- vs. BMI-trained model, $F_{1,45} = 5.31$,

p = .026; effect of Test Behaviour, $F_{2,90} = 3.33$, $p = .040$; no significant interaction. Monkey A: Cell 1 only: mean $r = 0.178$, s.e.m. = 0.021; Cell 1 + Cell 2: mean $r = 0.169$, s.e.m. = 0.017; Cell 1 – Cell 2: mean $r = 0.189$, s.e.m. = 0.019; two-way repeated-measures ANOVA: effect of torque- vs. BMI-Trained model, $F_{1,45} = 65.3$, $p < .001$; effect of Test Behaviour, $F_{2,90} = 1.91$, $p = .15$; no significant interaction).

Data in **Supplementary Fig. 8a,c** passed normality tests (Shapiro-Wilk, $p > .05$). In **Supplementary Fig 8b**, a transformation function, $Y' = ln(Y + 0.05)$, was first used, where *Y* is the original data and *Y'* is the transformed data, after which data passed normality tests (Shapiro Wilk, $p > .1$). The original data is plotted. In cases where Mauchly's Test of Sphericity was not passed ($p < .05$), degrees of freedom were adjusted appropriately by a Greenhouse-Geisser correction.

Overall we found no significant difference in our ability to estimate M1 and PMv neurons (**Supplementary Fig. 8c**; two-way independent samples ANOVA, effect of Area, $F_{1,88} = 0.016$, n.s.; effect of Monkey, $F_{1,88} = 0.181$, *n.s.*), although a significant interaction was found between factor Monkey and factor Area ($F_{1,88} = 6.78$, $p = .011$), with M1 neurons estimated better in Monkey D and PMv neurons better in Monkey A. We were able to estimate neurons with a wide range of firing rates, although performance increased slightly with mean firing rate (**Supplementary Fig. 8d**; regression line, *rho* = 0.23, $p = .03$).

In general, firing rate estimations based on data from the CED system performed less well than those with the TDT-based system. This was primarily because fewer *lf*-LFPs were available with the CED-based system, but also because the 1 Hz high-pass filtering of the LFPs recorded with the CED-based system removed some informative frequencies.

Trial-to-trial variation in firing rates and *lf*-LFP. (**Supplementary Fig. 9**). The 'cell-control' BMI task provided an additional opportunity to examine how the cell-LFP relationship revealed by the SRSP depends on neuronal firing rates and overt movement. In this example session, Monkey A performed 100 trials of a 1-D 'cell-control' task by modulating the firing rate of a single PMv neuron to acquire 4 different targets while the isometric torque generated by the left wrist was monitored (**Supplementary Fig. 9a**). Acquisition of the bottom target (reduced firing rate) was associated with wrist torque (**Supplementary Fig. 9e**), but the other targets were achieved without any consistent overt movement. STAs of the *lf*-LFP from a second PMv electrode were compiled using only spikes within ± 3 s of the end of successful trials for each target (**Supplementary Fig. 9d**; *red traces*). Although noisy due to the small number of events, a positive *lf*-LFP peak follows the trigger spike in each case, even for those targets which are not associated with the generation of torque (middle and top targets). Indeed the SRSP is remarkably consistent across targets with very different trial-averaged firing rate profiles (**Supplementary Fig. 9b**). This may seem counter-intuitive, especially since the trial-averaged *lf*-LFP does not show any obvious modulation for these targets (**Supplementary Fig. 9c**). Indeed, we can assess the SRSP that would be expected from task-related co-variation in firing rate and *lf*-LFP by calculating the cross-correlation between the trial-averaged firing rate and trial-averaged *lf*-LFP (**Supplementary Fig. 9d**; *green traces*). The peaks in these correlations are about an order of magnitude smaller than the true STA, suggesting that the SRSP does not merely reflect co-variation between signals that are time-locked to task events or wrist movements. Rather the SRSP includes correlations between firing rate and *lf*-LFP that vary trial-to-trial and therefore do not survive averaging aligned to task events.
